# Virulence factors and therapeutic methods of *Trueperella pyogenes*: A review

**DOI:** 10.1080/21505594.2025.2467161

**Published:** 2025-02-21

**Authors:** Xiangfu Wen, Jia Cheng, Mingchao Liu

**Affiliations:** a College of Veterinary Medicine, Hebei Agricultural University, Bao Ding, China; b Key Laboratory of Healthy Breeding in Dairy Cattle (Co-construction by Ministry and Province), Ministry of Agriculture and Rural Affairs, Hebei Agricultural University, Baoding, China

**Keywords:** Trueperella pyogenes, virulence factors, pathological symptoms, antimicrobial resistance, therapeutic strategies

## Abstract

*Trueperella pyogenes* is a prevalent opportunistic pathogen responsible for a wide range of infections in livestock and wildlife, such as in cattle, pigs, European bison and forest musk deer. Much of the successful infection of *T. pyogenes* relies on its virulence factors, including pyolysin as well as adhesion factors. The swift rise of bacterial resistance has highlighted the urgent need for developing new therapeutic strategies. Currently, virulence factor-mediated vaccine development and other therapeutic approaches are widely regarded as the primary interventions for addressing diseases associated with this pathogen. This review examines the broader virulence potential of *T. pyogenes*, focusing on haemolysin, host cell adhesion proteins, the prevalence of antibiotic resistance, and the development of vaccines mediated by virulence factors. Additionally, it discusses current and future approaches aimed at improving therapeutic interventions.

## Introduction

The pervasive presence of *Trueperella pyogenes* and other Gram-positive bacteria in both livestock hosts and external environments underscores their capacity for colonization, pathogenesis in pyogenic infections, and remarkable adaptation to extreme environmental conditions. Moreover, these organisms exhibit sophisticated mechanisms for modulating host immune responses, thereby enabling them to evade detection by host immune defence systems [1–3]. Furthermore, human infections can occur, particularly among immunocompromised individuals and those in close contact with livestock and their environments, which highlights the zoonotic potential of this microorganism [4,5]. The pathogenic potential of *T. pyogenes* is determined by several putative virulence factors. Pyolysin (Plo) is a major virulence factor, which is responsible for host cell lysis, and *plo* is found in all *T. pyogenes* strains [6,7]. Four fimbriae proteins (Fims), such as fimA, fimC, fimE and fimG, are related to adhesion, colonization, and pathogenesis of this gram-positive pathogen [8]. Neuraminidases, another virulence factor coded by *nanH* and *nanP*, promote the adhesion of *T. pyogenes* to the receptors of cryptic hosts and mucosal epithelial cells, with an enzymatic role in the catabolism of sialic acid [9]. These virulence factors are produced by both Gram-positive and Gram-negative pathogens and promote colonization, reduce mucosal viscosity, and weaken the immune response [9–11]. Environmental factors, host immune status, and bacterial virulence factors may interact to influence the occurrence and severity of infections [12]. This complexity complicates the control measures against this bacterium and has sparked discussions regarding its pathogenicity and modes of transmission [13,14].

Significant extent of drug resistance in *T. pyogenes* has been reported, with particularly high resistance to antimicrobials, including tetracycline, erythromycin, and clindamycin, observed across different regions [15,16] (Table 1). Different strategies for developing vaccines against *T. pyogenes* have been planned, including attenuated vaccines, DNA vaccines, and recombinant protein vaccines, and immune responses triggered by these vaccines and their protective efficacy have been tested in animal models [17–19]. Moreover, the integration of Chinese herbal medicines with several newly identified cytokines has introduced novel concepts and potential treatment approaches for diseases associated with this bacterium [20].

**Table 1.** Drug resistance rate of *T. pyogenes* in five countries and regions [113,135,149–153]. “-” means not tested.

Antimicrobials	Southern Kyushu, Japan (*n* = 167)	Columbia, USA (*n* = 22)	Tehran, Iran (*n* = 68)	Jilin Province, China (*n* = 27)	Northern China (*n* = 50)	Poland (*n* = 97)	Spain (*n* = 96)
Tetracycline	53.3%	–	26.5%	–	30%	43.3%	–
Erythromycin	13.8%	–	26.7%	85.2%	56%	7.2%	22.8%
Streptomycin	49.7%	–	–	–	–	–	–
Clindamycin	13.2%	–	–	–	50%	8.2%	–
Amikacin	0%	–	–	74.1%	–	–	–
Oxytetracycline	–	77%	–	–	–	–	–
Tylosin	–	18%	23.5%	–	–	–	–
Trimethoprim-sulfamethoxazol	–	–	70.6%	–	10%	–	92.7%
penicillin	–	–	–	0	–	–	6.3%
Enrofloxacin	–	–	16%	–	80%	40.2%	–

Exploration of the pathogenic mechanisms of *T. pyogenes* is important for identifying and optimizing therapeutic measures to reduce infections and maximize the quality of life of ill animals [21–23]. However, the pathogenic mechanisms of *T. pyogenes* are not yet fully understood, particularly under varying environmental and host conditions [24]. Therefore, this review aims to provide an overview of recent insights into the pathogenicity, virulence factors, and antibiotic resistance of *T. pyogenes*. In addition, this review discusses the methodologies for virulence factor-mediated vaccine development against *T. pyogenes* and outlines therapeutic strategies that can be employed to tackle the diseases associated with this bacterium, thereby providing new possibilities and scientific foundations for preventing and treating infections caused by *T. pyogenes*.

## Prevalence of *T. pyogenes* infections in different hosts

The initial documented observations of *T. pyogenes* date back to 1946 [25]. By 1974, epidemiological data revealed its association with approximately 66% incidents of bovine abortion [26]. A close association between *T. pyogenes*, and abortion and orchitis in livestock has been demonstrated, highlighting its critical role in reproductive health [27]. A microbiome analysis of bovine clinical endometritis has identified *T. pyogenes* as the primary causative pathogen [28]. In addition to affecting reproductive health, *T. pyogenes* is frequently implicated in mastitis with high clinical severity. It has been identified in 35% mastitis samples from cows aged <2 years [29–32]. The effects of the pathogen extend to the respiratory, urinary, and visceral systems of cattle [33,34]. *T. pyogenes* has been found to be responsible for 5% cases of urinary tract infections in calves aged 7 days to 3 months [35]. Additionally, it has been isolated from the kidneys of 7 out of 21 heifers with nephritis in a cohort of 2,426 slaughtered individuals [36]. Notably, *T. pyogenes* can also be found in asymptomatic cattle with detection rates of 51%, 17%, 19%, 8%, and 6% in tonsil, vaginal, calf, conjunctival sac, and nasal samples, respectively [37,38]. In small ruminants, *T. pyogenes* plays major roles in mastitis and endometritis, both of which significantly affect livestock productivity [39]. Its ability to cause severe infections highlights its importance as a pathogen in these animals. *T. pyogenes* has been linked to joint infections in piglets 21–25 days after farrowing, further demonstrating its pathological potential and broad impact across livestock species [40]. Furthermore, *T. pyogenes* has been identified in cases of foot dermatitis in chickens, highlighting its potential to cause significant morbidity in poultry [41]. Among the wildlife species, *T. pyogenes* is prevalent and has been primarily associated with cranial abscesses in white-tailed deer. For instance, it can penetrate intracranially, leading to a mortality rate as high as 35% in free-ranging adult males [42–44]. This indicates its widespread presence and pathogenic potential in wildlife.

Although rare, *T. pyogenes* has also been associated with human infections. It has been detected in leg ulcers among 495 school children in Thailand, suggesting its zoonotic potential and global presence [45].

Overall, *T. pyogenes* poses multiple risks to livestock, poultry, wildlife, and humans. Its association with abortion, orchitis, endometritis, mastitis, foot dermatitis, and respiratory diseases underscores its significant impact on animal productivity and health. In wild animals, its role in cranial abscesses further highlights its broad ecological presence. Collectively, these findings emphasize the urgent need for targeted prevention and management strategies for mitigating its impact across species, including the zoonotic risks posed to humans [39,41,46–51] (Table 2).

**Table 2.** Isolation rates of *T. pyogenes* in different hosts.

Sample source	Samples’ number	Related diseases	Detection time	Separation rate (%)	References
Forest musk deer samples	28	Abscesses	2011	100.00	Zhao et al., [48]
Milk samples	55	Mastitis	2009–2012	103.64	Nagib et al., [50]
Table egg layers	124	Pododermatitis	2015	1.80	Heidemann et al., [41]
Male white-tailed deers	65	Active cranial abscess	2015	70.70	Cohen et al., [47]
Domestic species	34	Nonrepetitive neurologic	2005–2021	8.80	Ribeiro et al., [49]
Postweaning pigs bodies	178	Lameness and swollen joints	2022	8.20	Salogni et al., [46]
European Bison	16	Abscesses	2022	14.6	Kwiecien et al., [51]
Small ruminants samples	316	Abscesses	2021–2023	27.2	Wei et al., [39]

### Cows

**Figure 1.**
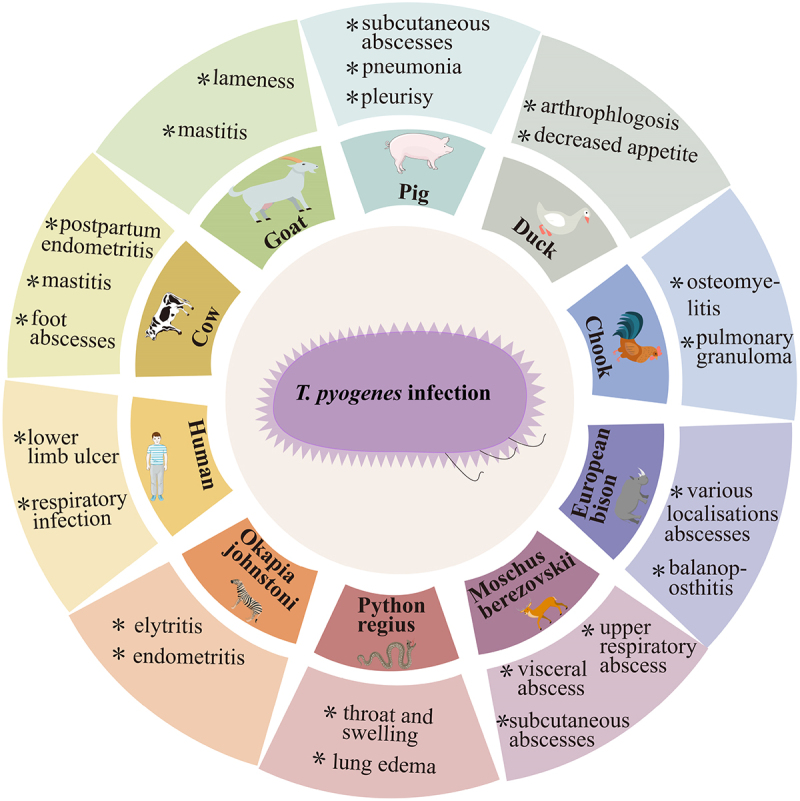
*T. pyogenes* infection symptoms in various animal species and humans.

Common diseases associated with *T. pyogenes* infection in dairy cattle include postpartum endometritis and mastitis [52]. Clinically, cases of *T. pyogenes*-induced foot abscesses in beef cattle have been documented [53] (Figure 1). Infection of *T. pyogenes* can lead to abnormalities in the oestrous cycle such as shortened intervals between oestrus cycles and the inability to exhibit oestrus. Affected cows often present with purulent vaginal discharge and elevated body temperature, and may also exhibit reduced milk yield, reddened and swollen udders, or reluctance to lactate owing to discomfort. Additional symptoms observed during clinical diagnosis include reduced appetite and lethargy [54,55].

### Pigs

Pigs are particularly susceptible to *T. pyogenes* infection. Infected pigs frequently develop subcutaneous abscesses, pneumonia, and pleurisy [56] (Figure 1). An investigation of pathogen isolation from lameness lesions in 22 infected pigs has revealed the presence of *T. pyogenes* in 73% samples [57]. Additionally, the immunocompromised state of affected pigs predisposes them to secondary infections with immunosuppressive diseases such as porcine reproductive and respiratory syndrome and porcine circovirus disease [58]. Failure to promptly treat infections in pigs can lead to the progression of septicaemia, potentially resulting in substantial economic loss for farmers [59].

### Goats

Goats and sheep harbour *T. pyogenes* in various diseases. *T. pyogenes* has been isolated from goats with mastitis, suggesting that this pathogen plays an important role in udder infections of goats [30]. Between 2015–, 104 *T. pyogenes* isolates have been obtained from 1,146 clinical samples [60]. These samples were collected from various body parts of infected sheep, including the udder, vagina, lymph nodes, and lungs. *T. pyogenes* is associated with lameness in migratory sheep and goats. Of 216 samples of foot lesion, 20.37% have been tested positive for *T. pyogenes* [61].

### Wildlife species

*T. pyogenes* has been documented as an infectious agent in wildlife species including forest musk deer and European bison. It has been identified in approximately all septic lesions of forest musk deer [62]. Infections caused by *T. pyogenes* in forest musk deer typically lead to subcutaneous abscesses and suppurative lesions in various internal organs; however, these infections generally do not result in mortality [63]. Compromised immunity, may facilitate secondary or concurrent infections by highly virulent pathogens, such as *Pseudomonas aeruginosa*, which could ultimately be the primary cause of mortality in forest musk deer [64]. Concurrently, these pathogens have been acknowledged as the primary aetiological agents of septic lesions in the upper respiratory tract of forest musk deer [65]. In European bison, abscesses have been observed at various locations, and *T. pyogenes* has been identified as an aetiological agent responsible for chronic necrotizing inflammation of the prepuce and penis, a condition known as balanoposthitis [51].

Additionally, *T. pyogenes* has been isolated from python (*Python regius*) and okapi (*Okapia johnstoni*). In a zoo setting, post-mortem examination of a python revealed a 15-cm swelling in the throat and head region, with *T. pyogenes* identified as the causative agent, which led to the death of the python. A substantial number of *T. pyogenes* has been detected in vaginal secretions of deceased okapi [66]. A northern greater galago (*Otolemur garnettii*) died from wound infections caused by fighting during oestrus, with necropsy revealing abscesses in the thoracic and abdominal cavities, which were attributed to *T. pyogenes* infection [67]. Additionally, three grey slender lorises in the Frankfurt Zoo, Germany suffered from severe septicaemia and died owing to complications Caused by *T. pyogenes* [68]. *T. pyogenes* is capable of causing brain abscesses, as it has been isolated from brain abscesses in roe deer (*Capreolus capreolus*) and white-tailed deer (*Odocoileus virginianus*); however, the exact route of infection remains unclear [46,69]. These findings highlight the potential of *T. pyogenes* to cause severe infections in various animal species, with a broad range of clinical manifestations.

### Poultry

The prevalence of *T. pyogenes* in poultry is relatively low; however, this bacterium has been isolated from the spleen, lungs, and air sacs of an aged rooster that succumbed to osteomyelitis [70]. Infection of *T. pyogenes* leads to symptoms, such as lethargy, reduced appetite, and inflamed and swollen joints, in affected ducklings [71]. Autopsy has revealed pus in the joint cavities and peptone-like changes in leg muscles of the affected ducks, with concurrent enlargement of the spleen and thymus. However, unlike that in other poultry species, infection of *T. pyogenes* in ducks does not typically result in mortality [72].

### Human

*pyogenes* has been classified as a zoonotic pathogen; however, documented cases of human infection by this bacterium remain scarce [73]. In Thailand, a case involving a patient with lower-limb ulcerative lesions has been attributed to *T. pyogenes* infection. Laboratory analyses have revealed that the patient had low titres of antibodies against *T. pyogenes*. Moreover, a radioimmunoassay demonstrated that *T. pyogenes* could be activated even in the absence of specific antibodies. It was hypothesized that the infection might have been transmitted by a fly coming in contact with the wound [74]. In the US, an individual acquired a respiratory infection following contact with a deer infected by *T. pyogenes* during a hunting expedition. The individual did not use respiratory protection while smoking during the hunt and subsequently developed *T. pyogenes* infection. The symptoms include dyspnoea, fatigue, constipation, and progressive onset of expectoration of orange-yellow sputum [75]. In both cases, the infections exhibited typical clinical features.

*pyogenes* is an uncommon cause of infection in humans, and current literature is restricted to case reports. Patients with intraabdominal infection, skin ulcer, sepsis, arthritis, pneumonia, and pyelitis have been reported [74,76–82]. Nine cases of infectious endocarditis have reported the isolation of *T. pyogenes*, of which three patients had a history of contact with farm animals, and five had underlying conditions that impaired their immune system [83–91] These cases suggest that both immunosuppression and contact with farm animals may serve as significant risk factors for *T. pyogenes* infection. In , the first reported case of head and neck infection caused by *T. pyogenes* in a human has further underscored the zoonotic potential of this bacterium [92].

## Virulence factors of *T. pyogenes*

The pathogenic potential of bacteria is typically closely associated with their virulence factors [93,94]. *T. pyogenes* harbour five major virulence factors such as Plo, collagen-binding protein (Cbp), neuraminidases (NanH and NanP), Fim, and biofilm [95,96]. These virulence factors are diverse in nature, and each fulfils distinct roles in the pathogenic process [97].

### Plo

Plo is the only identified exotoxin of *T. pyogenes* and is regarded as its paramount virulence factor, which has been detected in all analysed *T. pyogenes* strains [98,99] (Table 3). Plo belongs to the cholesterol-dependent cytolysin family [100] and consists of 524 amino acids with a molecular weight of 57.9 kDa. The toxin is intolerant to heat, strong acids, and bases, and demonstrates sensitivity to proteases such as trypsin and amylase [101]. Plo establishes β-barrel channels in the cell membrane by specifically binding to cholesterol. This interaction induces the efflux of K^+^ ions, leading to nucleotide-binding domain, leucine-rich repeat, and pyrin domain-containing protein 3 (NLRP3) inflammasome-mediated activation of the cysteine protease caspase-1 and subsequent cleavage of gasdermin D. These effects ultimately result in cellular pyroptosis [102]. During macrophage pyroptosis, the inflammatory cytokine interleukin (IL)-1β is released in high amount and initiates an immune response [103]. Additionally, disruption of the cell membrane further facilitates the release of intracellular nutrients, thereby providing a favourable environment for the proliferation of *T. pyogenes* [104].

**Table 3.** The detection rate of common genotypes of *T. pyogenes* in different sources. “-” means it was tested but not detected[9,10,47,99,105–111].

Animal species	Sample source	Strains’ number	*Plo* (%)	*NanH* (%)	*NanP* (%)	*FimA* (%)	*FimC* (%)	*FimE* (%)	*FimG* (%)	*CbpA* (%)	References
Livestock	Dairy cows breast	55	100	100	100	100	76	76	84	73	Ashrafi et al., [215]
	Dairy cows breast	89	100	52	44	100	76	91	18	21	Zastempowska et al., [109]
	Clinical bovine breast	45	100	40	75.6	100	88.9	86.6	6.7	35.6	Zheng et al., [111]
	Dairy cows body	68	100	100	100	100	68	76	71	91	Tamai et al., [106]
	Bovine septicaemia	100	100	56	62	98	80	95	11	11	Hideki et al., [107]
	Cattle uterus	65	100	83	76.9	100	69.2	76.9	61.5	56.9	Ashrafi et al., [10]
	Porcine lungs	27	100	25.9	51.9	100	29.6	59.3	0	66.7	Dong et al., [105]
	Porcine septicaemia	67	100	94	41.8	97	22.4	82.1	41.8	1.5	Hideki et al., [107]
	Cattle, goats, and sheep lesion	50	100	68	84	90	78	80	20	–	Rogovskyy et al., [9]
	Cattle, sheep, goats, dogs, equines, and a pig lesion	71	100	63.4	78.9	98.6	64.8	74.6	5.6	8.4	Risseti RM., [99]
Wildlife	Domestic and European bison lesion	97	100	66	72	100	76	–	62	9	Rzewuska et al., [108]
	European bison lesion	25	100	40	44	100	88	24	12	–	Rzewuska et al., [110]
	Male white-tailed deer lesion	66	100	79	100	100	80	98	70	12	Cohen BS et al., [47]

**Figure 2.**
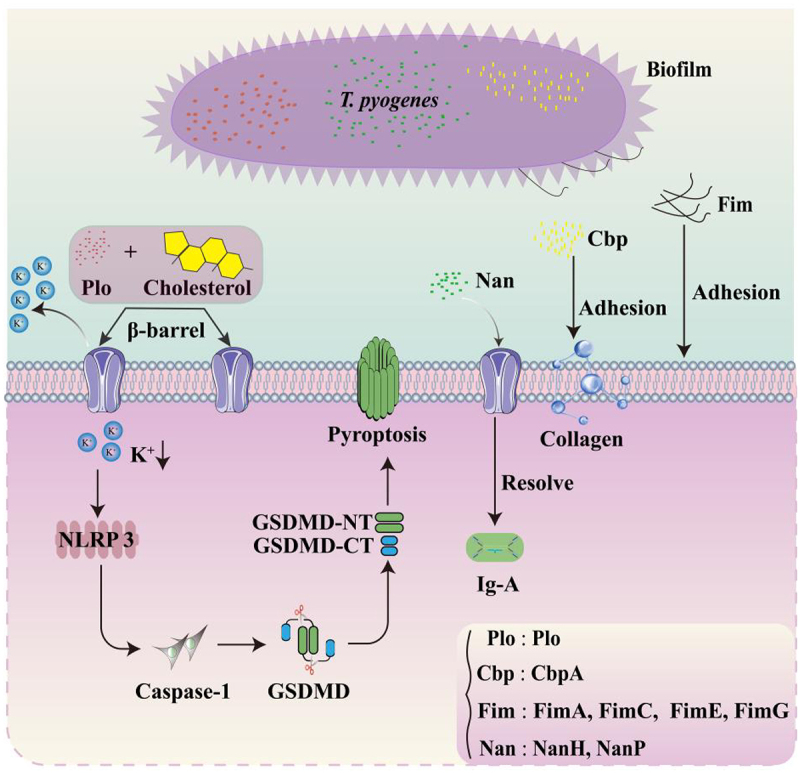
The action pathway of each virulence factor. The figure shows the genotypes of various virulence factors, including Plo, Cbp, Fim, Nan, and their roles in microbial pathogenic mechanisms. Specifically, Plo contributes to cellular membrane disruption via apoptotic pathways. Nan impair the host immune response by targeting and resolving IgA antibodies. Cbp facilitates the attachment and proliferation of *T. pyogenes* within host tissues through collagen binding. Additionally, Fim are crucial for bacterial adherence to host cells.

In addition to its well-established role in inducing cellular inflammatory responses, Plo contributes to an immunosuppressive environment that supports other pathogenic bacteria by modulating the cell signalling pathways [112]. Plo impairs the immune response induced by lipopolysaccharide and simultaneously aids in bacterial invasion in conjunction with lipoproteins, which are the virulence factors of *Escherichia coli* [113]. This interaction between Plo and lipoproteins is essential for the establishment and progression of infection, thereby highlighting the intricate mechanisms through which Plo enhances bacterial virulence [114]. Moreover, the 238th aspartic acid residue in Plo produced by *T. pyogenes* is critical for inducing inflammation and forming membrane pores [115]. The regulation of Plo expression, which involves multiple transcription factors and signalling pathways, is modulated by various factors, such as temperature, oxygen levels, and pH [116] (Figure 2).

### Cbp

Cbp is an extracellular matrix-binding protein synthesized by *T. pyogenes* and is classified within the microbial surface components recognizing adhesive matrix molecules (MSCRAMMs) protein family [117]. MSCRAMMs are a class of proteins that interact with host extracellular matrix components, thereby facilitating bacterial adhesion to the host [118]. Presently, *CbpA* is the only Cbp-expressing genotype identified among the virulence genotypes of *T. pyogenes*, exhibiting isolation rates of 73%, 91%, and 66.7% cases of clinical mastitis, abscesses, and porcine lungs [12,105,106]. The high isolation rate of *CbpA* is also evident from the data presented in Table 3.

Cbp interacts with host collagen primarily through its collagen-binding domain and establishes a stable interaction [119]. This interaction facilitates the colonization of *T. pyogenes* at the infection site and helps evade host immune clearance, thereby enhancing the persistence and severity of infection [120]. Cbp performs several critical biological functions in the pathogenesis of *T. pyogenes*. By binding to collagen, Cbp promotes the attachment and proliferation of *T. pyogenes* to host tissues, leading to infection [121]. Additionally, Cbps can induce the production of inflammatory factors and cytokines by activating cellular signalling pathways, which exacerbates the inflammatory response [122]. Moreover, Cbp interferes with the normal function of the host immune system and suppresses immune response, thereby increasing the insidious nature of an infection [123].

### Neuraminidases

*T. pyogenes* produces two primary neuraminidases, NanH and NanP encoded by *nanH* and *nanP*, with molecular weights of 107 kDa and 186.8 kDa, respectively [124]. These virulence genotypes are prevalent, with isolation rates of 25.9% and 51.9%, respectively, for *T. pyogenes* from porcine lungs [125]. In contrast, the other genotypes have been isolated at rates > 40%. Neuraminidases cleave sialic acid from receptors on the host cell surface, including glycoproteins, glycolipids, and carbohydrates, thereby facilitating bacterial adhesion [126,127]. In addition, these enzymes inhibit host immune response by exposing IgA antibodies, rendering them susceptible to cleavage by bacterial proteases [128]. These findings underscore the crucial role of neuraminidases in the pathogenesis of *T. pyogenes*.

### Fim

The cell surface of *T. pyogenes* is densely adorned with short, straight, and filamentous projections known as Fim [129,130]. Fims are observed exclusively via electron microscopy as they cannot be detected by light microscopy [60]. Fims are typically present in Gram-negative bacteria. *T. pyogenes* is a rare Gram-positive bacterium, which produce Fim [131]. These Fims exhibit proteinaceous and antigenic properties [46]. *T. pyogenes* encodes five distinct *fim* genes: *fimA*, *fimB*, *fimC*, *fimE*, and *fimG* [132].

Notably, *fimA* is expressed in nearly all *T. pyogenes* strains. *fimA* has been detected in 100% strains isolated from postpartum cows (Table 3). *fimC* and *fimG* are present in 67% of the strains, whereas *fimE* is present in 98% [133]. Coinfections involving *T. pyogenes* and *P. aeruginosa* are frequently observed in clinical settings [134]. *fimA* is present in all *P. aeruginosa* strains, whereas *fimC* and *fimE* were present in 29.6% and 59.3% strains, respectively (Table 3). Consequently, *FimA* is considered to be the predominant gene. Generally, the primary function of Fim is to facilitate bacterial adherence to host cells, a process not associated with bacterial motility; however, the specific mechanisms underlying this function are largely unexplored [135]. Furthermore, diminished expression of *fimA* may restrict the development of other Fims, whereas rapid upregulation of this gene can signify a transition from a symbiotic to a pathogenic state in *T. pyogenes* [136].

### Biofilm

A biofilm is a complex and dense matrix composed of proteins, lipids, DNA, and other extracellular polymers secreted by bacteria [137]. This structure enables bacteria to effectively resist host immune response and evade antibiotic penetration, and provides an optimal microenvironment for *T. pyogenes* during host colonization, facilitating bacterial growth and reproduction by supplying essential nutrients and water [138]. The formation of *T. pyogenes* biofilms is regulated by a two-component system, *PloS*/*PloR*, with *PloR* acting as an upregulator of biofilm formation. In a goat infection model, all *T. pyogenes* strains have been noticed to produce biofilms. Interestingly, different strains have exhibited varying intensities of biofilm formation [136]. Not all *T. pyogenes* strains produce biofilms. In a mastitis infection model in dairy cows, 90% *T. pyogenes* strains have formed biofilms [139]. During infection in captive forest musk deer, 94.4% *T. pyogenes* strains have formed biofilms [62]. These findings highlight the variability in biofilm formation among *T. pyogenes* strains across different host species and infection models, suggesting that biofilm formation plays a crucial role in the pathogen’s adaptability and persistence in diverse environments.

Extracellular DNA (eDNA) is an essential component of biofilms, and TatD DNase enhances biofilm stability by promoting eDNA release [140]. Biofilms further secrete antimicrobial substances that attenuate host immune response, thereby enhancing the capacity of bacteria to evade host immune defence [141]. Overall, biofilms of *T. pyogenes* serve multiple functions during host invasion, significantly enhancing the survival and proliferation of this bacterium [142]. However, during polymicrobial infections, biofilms of *T. pyogenes* may be disrupted by biomolecular substances secreted by the coinfecting bacteria [143].

## Therapeutic methods

The therapeutic strategies against *T. pyogenes* infections are still evolving. Therapeutic methods, including immunotherapy, biological agents, and bacteriophages, have shown promise. Nevertheless, these approaches require further investigation and rigorous experimental validation for establishing their efficacy and safety in clinical settings.

### Antimicrobials

In treating *T. pyogenes* and other pathogenic bacterial infections, antimicrobials remain the primary therapeutic approach owing to their efficacy in inhibiting bacterial growth and preventing disease progression. Therefore, antimicrobial susceptibility tests provide an empirical basis for clinical use of antimicrobial agents, avoiding therapeutic failure and possible development of antimicrobial resistance [144,145]. The broth microdilution method for determining the minimum inhibitory concentration (MIC) of any antimicrobial agent is recommended worldwide, and specific methods for testing infrequently isolated or fastidious bacteria have been recently published. The MIC distribution and genetic characteristics of 180 *T. pyogenes* isolates, obtained from slaughtered pigs reared under intensive and extensive farming practices, have been determined using MIC values and pulsed field gel electrophoresis. The 90% MIC values for penicillin, amoxycillin, ceftiofur, gentamicin, and enrofloxacin are low, suggesting that these antimicrobials can be optionally used to treat *T. pyogenes* infections [146]. The broth microdilution method has been used to determine the MIC of *T. pyogenes* isolated from the uteri of seven postpartum cow herds, showing high susceptibility of the bacterium to ticarcillin/clavulanic acid, ceftiofur, and enrofloxacin [147]. Furazolidone exhibits notable antibacterial effects against *T. pyogenes* and significantly enhances the survival rates in mice [148]. Collectively, these results emphasize the need for continuous research on antimicrobial efficacy and alternative treatments for enhancing therapeutic options and improving outcomes for infections caused by *T. pyogenes.*

The intensifying issues of antibiotic resistance and misuse of antibiotics have recently become a major focus of scientific investigation [149]. Bacterial resistance, in particular, has been a focus of extensive scrutiny. *T. pyogenes* demonstrates significant drug resistance, and inappropriate or prolonged antibiotic usage can exacerbate bacterial resistance [91]. Consequently, once effective, antimicrobials may gradually lose their efficacy, thereby complicating the therapeutic process. *T. pyogenes* strains isolated from 22 different farms have exhibited a 70.6% resistance rate to sulphonamide antimicrobials [150]. Furthermore, *T. pyogenes* has exhibited a 41% rate of multidrug resistance to antimicrobials [151].

Antibiotic monotherapy is inadequate for controlling bacterial infections [152]. Table 1 presents the regional statistics on antibiotic resistance in purulent secretions across six different areas [105,139,107,108,153–155]. The administration of antimicrobials for treating *T. pyogenes* infections can disrupt the natural balance of bacterial flora, leading to the proliferation of harmful bacteria and emergence of new infections, a phenomenon commonly known as superinfection [156]. This can increase the complexity and risks associated with treatment. Consequently, identification of novel therapeutic strategies to control bacterial infections is highly necessary [157].

### Vaccine

Considering the escalating antibiotic resistance exhibited by *T. pyogenes*, vaccine development has emerged as a pivotal focus in the ongoing battle against this bacterial pathogen [158]. A critical strategy in vaccine development involves exploiting the bacterium’s virulence factors as antigenic targets, which can elicit a robust immune response in the host, thereby preventing or attenuating bacterial infections [159]. This approach is based on several key attributes of the virulence factors; they play an indispensable role during bacterial infection, are frequent and highly specific bacterial proteins or polysaccharides, and typically induce a potent immune response in the host [160]. Proteomic tools have been developed to identify novel antigens with the potential to serve as putative vaccines [161,162]. Recently, several surface proteins have been tested as vaccine candidates for controlling infectious diseases, some of which have shown protective effects [163,164].

**Figure 3.**
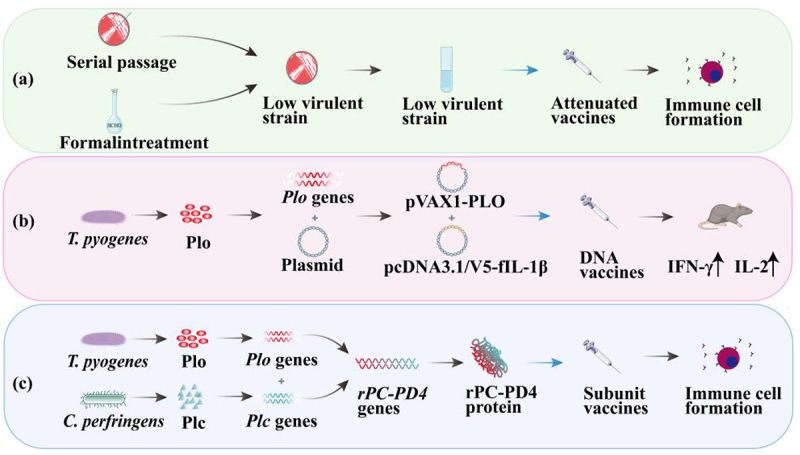
Overview of *T. pyogenes* Vaccine Development Strategies. The figure illustrates various vaccine development approaches against *T. pyogenes*, including attenuated vaccines derived from low-virulence strains obtained through formalin treatment and serial passage; DNA vaccines constructed using the *Plo* gene and plasmids, which induce high levels of ifn-γ and IL-2 in mice, primarily triggering a Th1 immune response; and recombinant protein vaccines combining virulence factor genes from *T. pyogenes* and *Clostridium perfringens*. These strategies highlight the immune responses and protective efficacy of different vaccine types.

Plo is a critical virulence factor expressed by *T. pyogenes* and is a promising target vaccine development against *T. pyogenes* [165]. Plo has been examined by treating the supernatant of *T. pyogenes* cultures with formalin and subsequently adsorbing the supernatant components onto aluminium hydroxide to synthesize a vaccine [166]. However, the slow growth of *T. pyogenes* typically necessitates the supplementation of animal serum to the culture medium for supporting proliferation. Consequently, this method is time-intensive and cost-prohibitive [167]. Vaccines developed using this technique frequently contain a mixture of protein components, which could potentially lead to biosafety issues or adverse effects [168]. Moreover, the serum components present in the vaccine can potentially transmit certain infectious agents, such as prions responsible for mad cow disease, or induce hypersensitivity reactions in animals [169]. Following the identification of *Plo*, subsequent studies have employed recombinant Plo (rPlo) expressed in prokaryotic systems for vaccine development [170,171]. However, rPlo demonstrates significant cytotoxicity and the potential to induce tissue damage. Detoxification of rPlo using formaldehyde represents a viable solution [171]; nonetheless, this process requires meticulous optimization for effective inactivation while preventing excessive denaturation of the antigen molecule, which may otherwise compromise its immunogenicity. An alternative strategy involves the utilization of Plo mutants with reduced cytotoxicity or truncated Plo molecules for vaccine development. Genetically detoxified Plo confers complete immune protection to mice [172]. Murine models receiving a chimeric antigen expressed in prokaryotic systems, incorporating the fourth domain of Plo molecule, have demonstrated partial protection against *T. pyogenes* challenge [173] (Figure 3). The proteomic “shaving” methodology has enabled the identification of 16 candidate proteins with potential for further development of vaccines and diagnostic tools, highlighting Plo as a highly promising antigenic target [174].

Furthermore, the genome of *T. pyogenes* contains Fims, particularly FimC and FimE, which are closely associated with the occurrence of uterine diseases in cows. After *T. pyogenes* infection, these Fims can induce the production of high levels of serum antibodies, specifically in cows with clinical endometritis [130]. These findings provide important potential targets for developing vaccines against bovine uterine diseases.

Genetic immunization is a promising strategy for eliciting protective immune responses against a broad spectrum of bacterial pathogens [175–178]. Compared to those of conventional live or subunit vaccines, DNA vaccines have several inherent advantages including ease of manufacturing, enhanced stability, and high safety profiles [179,180]. Coadministration of a recombinant plasmid, pVAX1-PLO constructed using *Plo* from *T. pyogenes*, with a recombinant plasmid encoding IL-1β in mice has demonstrated elevated levels of interferon gamma and IL-2, predominantly inducing T helper type 1 immune response, which has effectively reduced bacterial loads in the liver and peritoneal cavity, thereby increasing the survival rate of mice following *T. pyogenes* infection [181] (Figure 3).

Multiple pathogens commonly cooccur during bacterial infections, and virulence factors from diverse pathogens are frequently combined to formulate vaccines. A chimeric protein comprising the binding domains of *T. pyogenes* Plo and *Clostridium perfringens* phospholipase C offers partial protection against these two pathogens [174]. A protein structurally analogous to HtaA of *Corynebacterium diphtheriae* has been found to be highly expressed on the surface of *T. pyogenes*. Notably, the amino acid sequence of *T. pyogenes* HtaA is highly conserved across various *T. pyogenes* strains [182]. Subsequently, a nonhemolytic Plo mutant (PLOW497F), FimE, and a truncated cell wall protein (HtaA-2) have been used to develop both single-and multivalent protein vaccines, and their efficacies against lethal *T. pyogenes* challenge have been rigorously evaluated in mice. The findings suggest that PLOW497F and HtaA-2 hold significant promise for developing efficacious vaccines to prevent *T. pyogenes* infections [183]. The combined vaccine strategy has attracted considerable attention in the livestock industry primarily because of its potential to mitigate stress responses associated with the administration of multiple vaccines [184].

Despite extensive studies on vaccine development, the number of commercially available vaccines remains limited. Therefore, studies have focused on enhancing immune protection by implementing targeted vaccination programs. Heterologous prime-boost immunization strategy has been developed to control *T. pyogenes* infections [185]. This immunization approach integrates the pVAX1-PLO DNA vaccine with the His-PLO subunit vaccine, culminating in significantly enhanced humoral and cellular immune responses in the host. This novel therapeutic strategy holds great promise for developing future treatment modalities. Moreover, the *T. pyogenes* DNA vaccine has been encapsulated within chitosan nanoparticles, thereby enhancing the host’s resistance to *T. pyogenes* and setting a benchmark for future vaccine development [186]. Vaccination continues to serve as a cornerstone for preventing bacterial infection. In addition to inducing antibody production, vaccines can elicit robust T cell-mediated immune responses, thereby offering broad and much comprehensive protection [187]. Vaccine development has particularly focused on enhancing host immune memory by inducing long-lived memory T cells [188]. Memory T cells facilitate a rapid immune response during the early stages of infection and sustain long-term immune protection [189]. These findings open new avenues for developing effective vaccines against *T. pyogenes*.

### Natural products of plants

Various herbal compounds, including luteolin, tanshinones, chlorogenic acid, cinnamon, oregano, and thyme, have demonstrated inhibitory effects against *T. pyogenes* infection. The investigation of herbal remedies for impeding cellular invasion by *T. pyogenes* holds considerable significance [190]. Luteolin exerts multifaceted effects on *T. pyogenes*. It disrupts the integrity of the bacterial cell membrane, modulates protein expression, inhibits nucleic acid synthesis, and interferes with energy metabolism [191,192]. Moreover, luteolin can reverse the resistance of *T. pyogenes* to a broad range of antimicrobials. It enhances bacterial susceptibility to macrolides by inhibiting the activity of the MsrA efflux pump [193] and reduces gentamicin resistance by downregulating the expression of *MATE* that encodes multidrug and toxic compound efflux transporter proteins (MATEs) [194]. MATEs constitute a critical family of transporter proteins in living organisms [195], and their inhibition reduces the resistance of *T. pyogenes* to antimicrobials.

**Figure 4.**
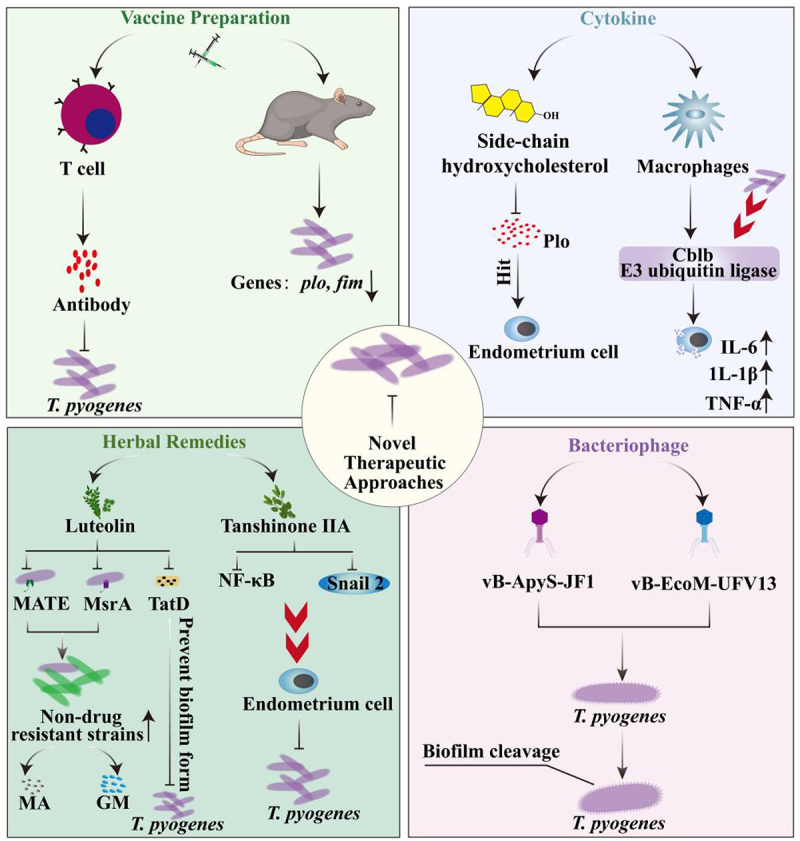
Treatment pathways for each treatment method. This figure shows the treatment against *T. pyogenes* infection. These methods include: Vaccine preparation: This method involves stimulation of lymphocytes to produce antibodies against *T. pyogenes*. Cytokine therapy: Specific cytokines such as E3 ubiquitin ligase Cblb and side-chain hydroxycholesterol were used to combat the infection of *T. pyogenes* by enhancing the ability of lymphocytes to release inflammatory factors and anti-virulence factor Plo. Phage therapy: This approach uses specific phages such as vB-ApyS-JF1 and vB-EcoM-UFV13 to target and destroy *T. pyogenes* by disrupting its membrane and structural proteins. Herbal therapies: Herbal compounds such as luteolin and tanshinones are used to inhibit bacterial multidrug resistance. They act by inhibiting proteins such as MATE and MsrA, thereby reducing the formation of gentamicin resistant strains. In addition, tanshinones by inhibiting Snail2 to prevent bacteria invasion of cells. Each of these approaches provides a unique mechanism to enhance existing therapies and address multidrug resistant strains of *T. pyogenes*.

TatD is instrumental in biofilm formation by *T. pyogenes*, and luteolin inhibits biofilm development by downregulating *TatD* expression [196]. In addition to luteolin, tanshinones protects cells from *T. pyogenes* infection. Tanshinone IIA effectively protects bovine endometrial epithelial cells from *E. coli* and *T. pyogenes* by inhibiting the activation of nuclear factor kappa beta (NF-κB) pathway and downregulating *Snail2* expression, thereby offering an approach to prevent bacterial infections [197]. A concise pathway for each treatment is shown in Figure 3. The efficacies of cinnamon, oregano, and thyme essential oils have also been investigated in countering infections caused by *T. pyogenes*. Cinnamon essential oil exerts a substantial therapeutic effect against infections caused by *T. pyogenes* and *E. coli* [198]. Chlorogenic acid, derived from honeysuckle, is effective in treating diseases caused by *T. pyogenes*. This substance activates the Kelch-like ECH-associated protein 1/nuclear factor erythroid 2-related factor 2 pathway while inhibiting NF-κB signalling, rendering it a promising candidate for treating endometritis caused by *T. Pyogenes* [199] (Figure 4). Collectively, these studies suggest that diverse natural compounds possess substantial potential to inhibit *T. pyogenes* infections and prevent associated diseases by modulating critical molecular targets and signalling pathways, thereby offering a robust scientific foundation for developing novel antibacterial therapeutic strategies.

### Other substances

Plo binds to cholesterol in the cell membrane to form pores that can lead to cell rupture. The dynamin inhibitor Dynasore protects cells by reducing cholesterol content in the cell membrane, thereby disrupting lipid raft structures and preventing pore formation [200,201]. Lipid rafts are membrane microdomains rich in cholesterol and other lipids, and several cellular signalling processes depend on these structures. The action of Dynasore is similar to that of another known cholesterol-lowering agent methyl-β-cyclodextrin that also protects cells from cytolysins such as Plo [100]. Additionally, mevalonate-derived isoprenoids increase cellular tolerance to Plo, which is associated with a reduction in cellular cholesterol levels and inhibition of the nuclear receptor subfamily 1 group H member 2 receptors [202].

Certain cytokines are capable of impeding the infection process during cell invasion by *T. pyogenes*. After *T. pyogenes* infection, a marked elevation in the expression of the E3 ubiquitin ligase Cblb has been observed. This upregulation triggers a robust inflammatory response and enhances macrophage-mediated phagocytosis in response to *T. pyogenes* infection. Inhibition of Cblb expression leads to a significant increase in bacterial load in mice, accompanied by a marked reduction in macrophage-mediated phagocytosis of *T. pyogenes*. These findings suggest that the E3 ubiquitin ligase Cblb plays a critical role in *T. pyogenes* infection [203].

The T4 phages vB-EcoM-UFV13 and vB-ApyS-JF1 are capable of inhibiting biofilm formation by *T. pyogenes*, thereby reducing its virulence [204]. This represents a highly cost-effective strategy for combating bacterial infections [205]. Furthermore, probiotics also exhibit therapeutic effects against *T. pyogenes* infections by inhibiting the adhesion of pathogenic bacteria through competition for cellular binding sites, thereby protecting tight-junction proteins and reducing cellular damage caused by *T. pyogenes* [206]. Additionally, probiotics can mitigate inflammatory damage to cells by inhibiting apoptosis-associated speck-like protein containing a caspase recruitment domain-dependent activation of the NLRP3 inflammasome. Probiotics show great potential as alternatives to antimicrobials [207]. Moreover, intracellular survival of *T. pyogenes* is significantly influenced by endoplasmic reticulum stress. Inhibition of all three branches of the unfolded protein response results in enhanced survival rates of *T. pyogenes* and diminished inflammatory responses. These findings provide novel intervention strategies for combating *T. pyogenes* infections [208].

Side-chain hydroxycholesterols play crucial roles in safeguarding endometrial cells from pathogenic bacterial invasion [209–211]. Specifically, 25-hydroxycholesterol and 27-hydroxycholesterol enhance the intrinsic defence mechanisms of endometrial cells against pore-forming toxins in dairy cows (Figure 4). Given that Plo produced by *T. pyogenes* primarily targets the host cell membrane, side-chain hydroxycholesterols may confer resistance to *T. pyogenes* [210]. Glucocorticoids can mitigate Plo-mediated damage to cell membranes [212]. Dexamethasone treatment reduces *Plo*-induced leakage of K^+^ ions and lactate dehydrogenase, limits alterations to the actin cytoskeleton, decreases blistering of the plasma membrane, and prevents cell lysis [213]. Similarly, hydrocortisone and fluticasone prevent septolysin-induced cellular damage [214].

## Conclusions

This review provides an overview of recent developments in our understanding of the pathogenicity, virulence factors, and antibiotic resistance of *T. pyogenes*. Its ability to withstand extreme environmental conditions and to modulate host immune responses allows it to evade host defences, thereby posing significant zoonotic risk, particularly in immunocompromised individuals. The pathogenicity of this bacterium is primarily driven by several key virulence factors. The incidence and severity of infections are modulated by complex interplay between environmental factors, host immune responses, and bacterial virulence mechanisms, significantly complicating the control of this pathogen and prompting substantial discourse regarding its pathogenic mechanisms and transmission dynamics. *T. pyogenes* has developed significant resistance to a spectrum of antimicrobials across diverse geographical regions. Therapeutic strategies, including vaccines designed to neutralize the virulence determinants of *T. pyogenes*, phytotherapies, and emerging cytokine-based treatments present promising avenues for controlling infections linked to this pathogen.

Additionally, reducing infections and improving the quality of life of affected animals depend on thorough investigation of the pathogenic mechanisms of *T. pyogenes* and optimization of therapeutic approaches, both of which are crucial. The pathogenic mechanisms of this bacterium have not yet been fully elucidated, particularly considering the variations in environmental and host contexts, which underscores the necessity of further in-depth studies.

## Data Availability

Data sharing is not applicable to this article as no new data were created or analysed in this study.

## References

[1] Foreyt WJ, Jessup DA. Fatal pneumonia of bighorn sheep following association with domestic sheep. J Wildl Dis. 1982;18(2):163–20. doi: 10.7589/0090-3558-18.2.163 7047767

[2] Aghamiri SM, Haghkhah M, Ahmadi MR, et al. Development of a multiplex PCR for the identification of major pathogenic bacteria of post-partum endometritis in dairy cows. Reprod Domest Anim. 2014;49(2):233–238. doi: 10.1111/rda.12259 24325777

[3] Schönecker L, Schnydrig P, Brodard I, et al. *Trueperella pecoris sp*. nov. isolated from bovine and porcine specimens. Int J Syst Evol Microbiol. 2021;71(6). doi: 10.1099/ijsem.0.004848 34161222

[4] Eisenberg T, Nagib S, Hijazin M, et al. *Trueperella pyogenes* as cause of a facial abscess in a grey slender loris (Loris lydekkerianus nordicus)–a case report. Berl Munch Tierarztl Wochenschr. 2012;125(9–10):407–410. doi: 10.2376/0005-9366-125-407 23045803

[5] Tamai IA, Mohammadzadeh A, Salehi TZ, et al. Expression of virulence factor genes in co-infections with *Trueperella pyogenes* isolates and other bacterial pathogens; an in vivo study. Microb Pathog. doi: 10.1016/j.micpath.2022.105435 35121072

[6] Zhang SH, Qiu JJ, Yang R, et al. Complete genome sequence of *Trueperella pyogenes*, isolated from infected farmland goats. Genome Ann-Ounc. 2016 Dec 15;4(6):e01421–16. doi: 10.1128/genomeA.01421-16 PMC5159590 27979957

[7] Reddy CA, Cornell CP, Kao M. Hemin-dependent growth stimulation and cytochrome synthesis in *Corynebacterim pyogenes*. J Bacteriol. 1977;130(2):965–967. doi: 10.1128/jb.130.2.965-967.1977 263823 PMC235308

[8] Fujimoto H, Shimoji N, Sunagawa T, et al. Differences in phenotypic and genetic characteristics of *Trueperella pyogenes* detected in slaughtered cattle and pigs with septicemia. J Vet Med Sci. 2020;82(5):626–631. doi: 10.1292/jvms.19-0370 32213728 PMC7273585

[9] Rogovskyy AS, Lawhon S, Kuczmanski K, et al. Phenotypic and genotypic characteristics of *Trueperella pyogenes* isolated from ruminants. J Vet Diagn Invest. 2018;30(3):348–353. doi: 10.1177/1040638718762479 29528808 PMC6505804

[10] Ashrafi Tamai I, Mohammadzadeh A, Ghalyanchi Langeroudi A, et al. Complete genome sequence of *Trueperella pyogenes* strain Arash114, isolated from the uterus of a water buffalo (Bubalus bubalis) in Iran. BMC Res Notes. 2021 Aug 23;14(1):323. doi: 10.1186/s13104-021-05734-1 34425879 PMC8381550

[11] Wente N, Leimbach S, Woudstra S, et al. *Trueperella pyogenes*—strain diversity and occurrence in dairy herds. Pathogens. 2024;13(7):534. doi: 10.3390/pathogens13070534 39057761 PMC11279676

[12] Mee JF. Invited review: bovine abortion-incidence, risk factors and causes. Reprod Domest Anim. 2023;58(Suppl 2):23–33. doi: 10.1111/rda.14366 37186386

[13] Maan MK, Chaudhry TH, Sattar A, et al. Dose optimization of aditoprim-sulfamethoxazole combinations against *Trueperella pyogenes* from patients with clinical endometritis by using semi-mechanistic PK/PD model. Front Pharmacol. 2021 Nov 16;12:753359. doi: 10.3389/fphar.2021.753359 34867364 PMC8635024

[14] Marchionatti E, Kittl S, Sendi P, et al. Whole genome-based antimicrobial resistance, virulence, and phylogenetic characteristics of *Trueperella pyogenes* clinical isolates from humans and animals. Vet Microbiol. 2024;294:110102. doi: 10.1016/j.vetmic.2024.110102 38749210

[15] Jost A, Sickinger M. Helcococcus ovis associated with septic arthritis and bursitis in calves - a case report. BMC Vet Res. 2021 Sep 3;17(1):291. doi: 10.1186/s12917-021-02996-6 34479562 PMC8414772

[16] Piersanti RL, Zimpel R, Molinari PCC, et al. A model of clinical endometritis in holstein heifers using pathogenic Escherichia coli and *Trueperella pyogenes*. J Dairy Sci. 2019;102(3):2686–2697. doi: 10.3168/jds.2018-15595 30692014 PMC6445275

[17] Stotz MK, Henry DD, Crossland WL. Evaluation of immunoglobulin-Y in place of tylosin phosphate in the diets fed to holstein steers and preliminary analysis of liver abscess duration on animal growth performance. Transl Anim Sci. 2020 Dec 5;5(1):txaa225. doi: 10.1093/tas/txaa225 33501416 PMC7810255

[18] Zhang W, Meng X, Wang J. Sensitive and rapid detection of *Trueperella pyogenes* using loop-mediated isothermal amplification method. J Microbiol Methods. 2013;93(2):124–126. doi: 10.1016/j.mimet.2013.03.002 23517678

[19] Sangiorgio DB, Hilty M, Kaiser-Thom S, et al. The influence of clinical severity and topical antimicrobial treatment on bacteriological culture and the micro-biota of equine pastern dermatitis. Vet Dermatol. 2021;32(2):173–e41. doi: 10.1111/vde.12912 33417744 PMC8048527

[20] Ali A, Derar DR. Clinical and subclinical endometritis in dromedary camels: an overview of definition and clinical presentation, etiopathogenesis, diagnostic biomarkers, and treatment protocols. Anim Reprod Sci. 2023;257:107328. doi: 10.1016/j.anireprosci.2023.107328 37683534

[21] Çeçen G, Buyukcangaz EK, Çalışkan ÜG, et al. INTERDIGITAL NECROB -ACILLOSIS ASSOCIATED with *Trueperella pyogenes* in GOITERED GAZELLES (GAZELLA SUBGUTTU ROSA). J Zoo Wildl Med. 2018;49(2):429–434. doi: 10.1638/2017-0050.1 29900794

[22] Kaura R, Dorbek-Kolin E, Loch M, et al. Association of clinical respiratory disease signs and lower respiratory tract bacterial pathogens with systemic inflammatory response in pre-weaned dairy calves. J Dairy Sci. 2024 Mar 22;107(8):5988–5999. doi: 10.3168/jds.2023-24084 38522828

[23] Skov RL, Sanden AK, Danchell VH, et al. Systemic and deep-seated infections caused by *Arcanobacterium haemolyticum*. Eur J Clin Microbiol Infect Dis. 1998;17(8):578–582. doi: 10.1007/BF01708624 9796659

[24] Thakur Z, Vaid RK, Anand T, et al. Comparative genome analysis of 19 *Trueperella pyogenes* strains originating from different animal species reveal a genetically diverse open pan-genome. Antibiotics (Basel). 2022;12(1):24. Published 2022 Dec 24. doi: 10.3390/antibiotics12010024 36671226 PMC9854608

[25] Maclean PD, Liebow AA, Rosenberg AA. A *hemolytic corynebacterium* resembling *Coryne-bacterium* ovis and *Corynebacterium pyogenes* in man. J Infect Dis. 1946;79(1):69–90. doi: 10.1093/infdis/79.1.69 20996930

[26] Sorensen GH. *Corynebacterium pyrog-enes*; a biochemical and serological study. Acta Vet Scand. 1974;15(4):544–554. doi: 10.1186/BF03547225 4218062 PMC8555158

[27] Samkange A, van der Westhuizen J, Voigts AS, et al. Investigation of the outbreaks of abortions and orchitis in livestock in Namibia during 2016–2018. Trop Anim Health Prod. 2022 [cited 2022 Oct 15];54(6):346. doi: 10.1007/s11250-022-03342-0 36242679

[28] Paiano RB, Moreno LZ, Gomes VTM, et al. Assessment of the main pathogens associated with clinical and subclinical endometritis in cows by culture and MALDI-TOF mass spectrometry identification. J Dairy Sci. 2022;105(4):3367–3376. doi: 10.3168/jds.2021-20642 35181136

[29] Slee KJ, McOrist S. Mastitis due to a group of pyogenic bacteria. Aust Vet J. 1985;62(2):63–65. doi: 10.1111/j.1751-0813.1985.tb14239.x 3888167

[30] Al-Graibawi MA, Sharma VK, Al-Shammari AJ. Microbial pathogens from goat mastitis and phage-typing of Staphylococcus aureus isolates. Comp Immunol Microbiol Infect Dis. 1986;9(1):23–28. doi: 10.1016/0147-9571(86)90071-8 2945693

[31] Van den Bogaard AE, Hazen MJ, Vecht U. The detection of obligate anaerobic bacteria in udder secretions of dry cattle with mastitis during summer: a comp-arison between gas-liquid chromatogra-phy and bacteriological culturing me-thods. Vet Microbiol. 1987;14(2):173–182. doi: 10.1016/0378-1135(87)90009-5 2444028

[32] Sørensen GH. Comparative studies on *Corynebacterium pyogenes* toxin form-ation in monocultures and mixed cultures. The demonstration of a stimulating effect of peptococcus indo-licus and stuart-schwan cocci. Acta Vet Scand. 1980;21(3):438–447. doi: 10.1186/BF03546876 7004149 PMC8317775

[33] Bitsch V, Friis NF, Krogh HV. A microbiological study of pneumonic calf lungs. Acta Vet Scand. 1976;17(1):32–42. doi: 10.1186/BF03547941 1266685 PMC8383976

[34] Yamamoto S, Okumura S, Kobayashi R, et al. Bovine respiratory syncytial virus enhances the attachment of *Trueperella pyogenes* to cells. J Vet Med Sci. 2024;86(10):1068–1075. doi: 10.1292/jvms.24-0068 39111845 PMC11442402

[35] Yeruham I, Elad D, Avidar Y, et al. Four-year survey of urinary tract infections in calves in Israel. Vet Rec. 2004;154(7):204–206. doi: 10.1136/vr.154.7.204 14994858

[36] Rosenbaum A, Guard CL, Njaa BL, et al. Slaughterhouse survey of pyelonephritis in dairy cows. Vet Rec. 2005;157(21):652–655. doi: 10.1136/vr.157.21.652 16299366

[37] Wagener K, Prunner I, Pothmann H, et al. Diversity and health status specific fluctuations of intrauterine microbial communities in postpartum dairy cows. Vet Microbiol. 2015;175(2–4):286–293. doi: 10.1016/j.vetmic.2014.11.017 25497238

[38] Sorensen GH. Studies on the occurrence of peptococcus indolicus and *Coryne-bacterium pyogenes* in apparently healthy cattle. Acta Vet Scand. 1976;17(1):15–24. doi: 10.1186/BF03547939 773138 PMC8383990

[39] Wei Y, Wang B, Wu K, et al. Prevalence, virulence genes, drug resistance and genetic evolution of *Trueperella pyogenes* in small ruminants in Western China. Animals (Basel). 2024 [cited 2024 Oct 14];14(20):2964. doi: 10.3390/ani14202964 39457894 PMC11503795

[40] Földi D, Nagy EZ, Tóth G, et al. *Mycoplasma hyopharyngis* isolated from the joint of a weaner: a case report. Acta Vet Hung. 2024 [cited 2024 Aug 29];72(3):155–160. doi: 10.1556/004.2024.01078 39213125

[41] Heidemann Olsen R, Christensen H, Kabell S, et al. Characterization of prevalent bacterial pathogens associated with pododermatitis in table egg layers. Avian Pathol. 2018;47(3):281–285. doi: 10.1080/03079457.2018.1440066 29517269

[42] Baumann CD, Davidson WR, Roscoe DE, et al. Intracranial abscessation in white-tailed deer of North America. J Wildl Dis. 2001;37(4):661–670. doi: 10.7589/0090-3558-37.4.661 11763729

[43] Davidson WR, Nettles VF, Hayes LE, et al. Epidemiologic features of an intracranial abscessation/suppurative me-ningoencephalitis. J Wildl Dis. 1990;26(4):460–467. doi: 10.7589/0090-3558-26.4.460 2250322

[44] Nettles VF, Quist CF, Lopez RR, et al. Morbidity and mortality factors in key deer (Odocoileus virgini-anus clavium). J Wildl Dis. 2002;38(4):685–692. doi: 10.7589/0090-3558-38.4.685 12528433

[45] Semaan A, Tayeh GA, Chebel JA, et al. *Arcanoba- cterium pyogenes* and encrustedpyeliti-s. Future Science OA. 6(1):FSO430[2024–07–20]. doi: 10.2144/fsoa-2019-0021 PMC6920744 31915531

[46] Salogni C, Capucchio MT, Colombino E, et al. Bacterial polyarthritis in post-weaning pigs in a high-density swine breeding area in Italy. J Vet Diagn Invest. 2022;34(4):709–711. doi: 10.1177/10406387221090903 35593676 PMC9266520

[47] Cohen BS, Belser EH, Keeler SP, et al. Isolation and genotypic characterization of *Trueperella* (*Arcanobacterium*) *pyogenes* recovered from active cranial abscess infections of male white-tailed deer (Odocoileus virginianus). J Zoo Wildl Med. 2015;46(1):62–67. doi: 10.1638/2014-0124R1.1 25831577

[48] Zhao KL, Liu Y, Zhang XY, et al. Detection and characterization of antibiotic-resistance genes in *Arcanobacterium pyogenes* strains from abscesses of forest musk deer. J Med Microbiol. 2011;60(Pt 12):1820–1826. doi: 10.1099/jmm.0.033332-0 21852523

[49] Ribeiro MG, Pereira TT, de Lima Paz PJ, et al. Bacterial identification in cerebrospinal fluid of domestic species with neurologic signs: a retrospective case-series study in 136 animals (2005–2021). Braz J Microbiol. 2023;54(1):449–457. doi: 10.1007/s42770-022-00891-2 36571673 PMC9944471

[50] Nagib S, Rau J, Sammra O, et al. Identification of *Trueperella pyogenes* isolated from bovine mastitis by fourier transform infrared spectroscopy. PLOS ONE. 2014 [cited 2014 Aug 18];9(8):e104654. doi: 10.1371/journal.pone.0104654 25133407 PMC4136790

[51] Kwiecień E, Stefańska I, Kizerwetter-Świda M, et al. Prevalence and genetic diversity of *Trueperella pyogenes* isolated from infections in European Bison (Bison bonasus). Animals (Basel). 2022 [cited 2022 Jul 18];12(14):1825. doi: 10.3390/ani12141825 35883372 PMC9311551

[52] Husnain A, Arshad U, Zimpel R, et al. Induced endometrial inflammation compromises conceptus development in dairy cattle†. Biol Reprod. 2023;109(4):415–431. doi: 10.1093/biolre/ioad088 37540198 PMC10577276

[53] Kontturi M, Junni R, Simojoki H, et al. Bacterial species associated with interdigital phlegmon outbreaks in finnish dairy herds. BMC Vet Res. 2019 [cited 2019 Jan 29];15(1):44. doi: 10.1186/s12917-019-1788-x 30696445 PMC6352363

[54] Silva JCC, Siqueira LC, Rodrigues MX, et al. Intrauterine infusion of a pathogenic bacterial cocktail is associated with the development of clinical metritis in postpartum multiparous holstein cows. J Dairy Sci. 2023;106(1):607–623. doi: 10.3168/jds.2022-21954 36400620

[55] Paiano RB, Bonilla J, Moreno AM, et al. Clinical endometritis with *Trueperella pyogenes* reduces reprod-uctive performance and milk production in dairy cows. Reprod Domest Anim. 2021;56(12):1536–1542. doi: 10.1111/rda.14017 34510600

[56] Qin L, Meng F, He H, et al. Inflammation plays a critical role in damage to the bronchiolar epithelium induced by *Trueperella pyogenes* in vitro and in vivo. Infect Immun. 2023;91(12):e0027323. doi: 10.1128/iai.00273-23 37929972 PMC10714949

[57] Engiles JB, Fanzone N, Wulster KB, et al. Gross, histopathologic, microbiologic, and radiologic characterization of lesions associated with clinical lameness in a cohort of group-housed sows euthanized for lameness. Vet Pathol. 2022;59(6):960–972. doi: 10.1177/03009858221114470 35938491

[58] Salogni C, Lazzaro M, Giovannini S, et al. Causes of swine polyserositis in a high-density breeding area in Italy. J Vet Diagn Invest. 2020;32(4):594–597. doi: 10.1177/1040638720928973 32495719 PMC7438655

[59] Poor AP, Moreno LZ, Monteiro MS, et al. Vaginal microbiota signatures in healthy and purulent vulvar discharge sows. Sci Rep. 2022 [cited 2022 Jun 1];12(1):9106. doi: 10.1038/s41598-022-13090-8 35650232 PMC9160009

[60] Kang HJ, Paterson NG, Gaspar AH, et al. The *Corynebacterium diphtheriae* shaft pilin SpaA is built of tandem Ig-like modules with stabilizing isopeptide and disulfide bonds [published correction appears in Proc Natl Acad Sci U S A. 2009 Oct 27;106(43): 18427]. Proc Natl Acad Sci U S A. 2009;106(40):16967–16971. doi: 10.1073/pnas.0906826106 19805181 PMC2761350

[61] Wani AH, Verma S, Sharma M, et al. Infectious lameness among migratory sheep and goats in north-west India, with particular focus on anaerobes. Rev Sci Tech. 2015;34(3):855–867. doi: 10.20506/rst.34.3.2401 27044157

[62] Zhao K, Tian Y, Yue B, et al. Virulence determinants and biofilm production among *Trueperella pyogenes* recovered from abscesses of captive forest musk deer. Arch Microbiol. 2013;195(3):203–209. doi: 10.1007/s00203-013-0869-7 23354327

[63] Zhao K, Ma J, Wang X, et al. Population divergence of *Pseudomonas aeruginosa* can lead to the coexistence with *Escherichia coli* in animal suppurative lesions. Vet Microbiol. 2019;231:169–176. doi: 10.1016/j.vetmic.2019.03.014 30955805

[64] Chen Q, Zhao K, Li H, et al. Antibacterial and anti-virulence effects of furazolidone on *Trueperella pyogenes* and *Pseudomonas aeruginosa*. BMC Vet Res. 2022 [cited 2022 Mar 24];18(1):114. doi: 10.1186/s12917-022-03216-5 35331229 PMC8943969

[65] Lu G, Wang Z, Zhang B, et al. Detecting forest musk deer abscess disease pathogens using 16S rRNA high-throughput sequencing technology. Animals (Basel). 2023 [cited 2023 Oct 8];13(19):3142. doi: 10.3390/ani13193142 37835748 PMC10572063

[66] Ahmed MFE, Alssahen M, Lämmler C, et al. Studies on *Trueperella pyogenes* isolated from an okapi (Okapia johnstoni) and a royal python (Python regius). BMC Vet Res. 2020 [cited 2020 Aug 14];16(1):292. doi: 10.1186/s12917-020-02508-y 32795301 PMC7427953

[67] Salleng KJ, Burton BJ, Apple TM, et al. Isolation of *Trueperella pyogenes* in a case of thoracic and abdominal abscess in a galago (Otolemur garnettii). J Med Primatol. 2016;45(4):198–201. doi: 10.1111/jmp.12223 27338233

[68] Nagib S, Glaeser SP, Eisenberg T, et al. Fatal infection in three grey slender lorises (Loris lydekkerianus nordicus) caused by clonally related *Trueperella pyogenes*. BMC Vet Res. 2017 [cited 2017 Aug 29];13(1):273. doi: 10.1186/s12917-017-1171-8 28851356 PMC5576266

[69] Wickhorst JP, Hassan AA, Sheet OH, et al. *Trueperella pyogenes* isolated from a brain abscess of an adult roebuck (Capreolus capreolus). Folia Microbiol (Praha). 2018;63(1):17–22. doi: 10.1007/s12223-017-0533-8 28534230

[70] da Silva AP, Shivaprasad HL, Jerry C, et al. An uncommon case of *Trueperella pyogenes* infection in an adult backyard rooster and a retrospective study; 2000–20. Avian Dis. 2021;65(1):171–176. doi: 10.1637/avian-diseases-D-20-00125 34339137

[71] Olson ME, Ceri H, Morck DW, et al. Biofilm bacteria: formation and comparative susceptibility to antibiotics. Can J Vet Res. 2002;66(2):86–92. doi: 10.1080/03079450120118685 11989739 PMC226988

[72] Giron TV, Vieira BS, Viott AM, et al. Mechanical removal (epidermal scarification) of pododermatitis injuries reduces the presence of both inflammatory tissue and its associated microbiota in broiler feet. Poult Sci. 2019;98(3):1455–1460. doi: 10.3382/ps/pey497 30325460

[73] Gahrn-Hansen B, Frederiksen W. Human infections with *Actinomyces pyogenes* (*Corynebacterium pyogenes*). Diagn Microbiol Infect Dis. 1992;15(4):349–354. doi: 10.1016/0732-8893(92)90022-l 1611850

[74] Kotrajaras R, Tagami H. *Corynebacter-ium pyogenes*. Its pathogenic mechanism in epidemic leg ulcers in Thailand. Int J Dermatol. 1987;26(1):45–50. doi: 10.1111/j.1365-4362.1987.tb04575.x 3549593

[75] Meili Z. *Trueperella pyogenes* pharyngitis in an immunocompetent 40-year-old man. BMJ Case Rep. 2020 [cited 2020 Nov 23];13(11):e236129. doi: 10.1136/bcr-2020-236129 PMC7684657 33229475

[76] Levy CE, Pedro RJ, Von Nowakonski A, et al. *Arcanobacterium pyoge*nes Sepsis in farmer, Brazil. Emerg Infect Dis. 2009;15(7):1131. doi: 10.3201/eid1507.081072 19624940 PMC2744241

[77] Hermida Amejeiras A, Romero Jung P, Cabarcos Ortiz De Barrón A, et al. A propósito de un caso de neumonía por *Arcanobacterium pyogenes* [One case of pneumonia with *Arcanobacterium pyogenes*]. An Med Interna. 2004;21(7):334–336. doi: 10.4321/s0212-71992004000700006 15347239

[78] Kavitha K, Latha R, Udayashankar C, et al. Three cases of *Arcanobacterium pyogenes*-associated soft tissue Infection. J Med Microbiol. 2010;59(6):736–739. doi: 10.1099/jmm.0.016485-0 20299502

[79] Drancourt M, Oulès O, Bouche V, et al. Two cases of *Actinomyces pyogenes* infection in humans. Eur J Clin Microbiol Infect Dis. 1993;12(1):55–57. doi: 10.1007/BF01997060 8462565

[80] Lynch M, O’Leary J, Murnaghan D, et al. *Actinomyces pyogenes* septic arthritis in a diabetic farmer. J Infect. 1998;37(1):71–73. doi: 10.1016/S0163-4453(98)90862-3 9733386

[81] Nicholson P, Kiely P, Street J, et al. Septic arthritis due to *Actinomyces pyogenes*. Injury. 1998;29(8):640–642. doi: 10.1016/s0020-1383(98)00142-9 10209601

[82] Semaan A, Tayeh GA, Chebel JA, et al. *Arcanobacterium pyogenes* and encrusted pyelitis. Future Sci OA. 2019;6(1):Fso430. doi: 10.2144/fsoa-2019-0021 31915531 PMC6920744

[83] Zhang H, Shi Z, Yang Q, et al. Endocarditis caused by *Arcanobacterium pyogenes*. Chin Med J (Engl). 2014;127(19):3510–3511. doi: 10.3760/cma.j.issn.0366-6999.20133302 25269923

[84] Jootar P, Gherunpong V, Saitanu K. *Corynebacterium pyogenes* endocarditis. Report of a case with necropsy and review of the literature. J Med Assoc Thai. 1978;61(10):596–601. 361919

[85] Reddy I, Ferguson DA Jr, Sarubbi FA. Endocarditis due to *Actinomyces pyogenes*. Clin Infect Dis. 1997;25(6):1476–1477. doi: 10.1086/516992 9431403

[86] Gómez-Mateos J, Ubeda A, Florez C, et al. Endocarditis due to *Arcanobacterium pyogenes*: the first case in Europe. Enferm Infecc Microbiol Clin. 2009;27(4):251–252. doi: 10.1016/j.eimc.2008.04.009 19249133

[87] Chesdachai S, Larbcharoensub N, Chansoon T, et al. *Arcanobacterium pyogenes* endocarditis: a case report and literature review. Southeast Asian J Trop Med Public Health. 2014;45(1):142–148. doi: 10.1017/S1368980013000086 24964663

[88] Tang ASO, Leong TS, Tan R, et al. Thrombotic thrombocytopenic purpura-like syndrome associated with *Arcanobacterium pyogenes* endocarditis in a post-transplant patient: a case report. Med J Malaysia. 2018;73(5):345. 30350823

[89] Plamondon M, Martinez G, Raynal L, et al. A fatal case of *Arcanobacterium pyogenes* endocarditis in a man with no identified animal contact: case report and review of the literature. Eur J Clin Microbiol Infect Dis. 2007;26(9):663–666. doi: 10.1007/s10096-007-0354-9 17610093

[90] Deliwala S, Beere T, Samji V, et al. When zoonotic organisms cross over-*Trueperella pyogenes* endocarditis presenting as a septic embolic stroke. Cureus. 2020;12(4):e7740. doi: 10.7759/cureus.7740 32455060 PMC7241225

[91] Stuby J, Lardelli P, Thurnheer CM, et al. *Trueperella pyogenes* endocarditis in a swiss farmer: a case report and review of the literature. BMC Infect Dis. 2023 [cited 2023 Nov 23];23(1):821. doi: 10.1186/s12879-023-08810-y 37996784 PMC10668470

[92] Calo’ L, Scarano E, Brigato F, et al. Bilateral cavernous sinus and ophthalmic vein thrombosis secondary to *Trueperella pyogenes* rhinosinusitis. Indian J Otolaryngol Head Neck Surg. 2024;76(3):2840–2843. doi: 10.1007/s12070-024-04505-1 38883471 PMC11169137

[93] Ramírez-Ordorica A, Patiño-Medina JA, Meza-Carmen V, et al. Volatile fingerprint mediates yeast-to-mycelial conversion in two strains of beauveria bassiana exhibiting varied virulence. J Fungi (Basel). 2023 [cited 2023 Nov 24];9(12):1135. doi: 10.3390/jof9121135 38132736 PMC10744692

[94] Jonker A, Thompson PN, Michel AL. Approaches to increase recovery of bacterial and fungal abortion agents in domestic ruminants. Onderstepoort J Vet Res. 2023 [cited 2023 Jan 11];90(1):e1–e10. doi: 10.4102/ojvr.v90i1.2010 PMC9900296 36744493

[95] Zhou Y, Shao Z, Dai G, et al. Pathogenic infection characteristics and risk factors for bovine respiratory disease complex based on the detection of lung pathogens in dead cattle in Northeast China. J Dairy Sci. 2023;106(1):589–606. doi: 10.3168/jds.2022-21929 36333140

[96] Machado VS, Bicalho R. Genome Announc. 2014;2(2). doi: 10.1128/genomeA.00400-14 PMC4007991 24786956

[97] Carusi J, Kabuki DY, de Seixas Pereira PM, et al. *Aeromonas spp*. In drinking water and food: occurrence, virulence potential and antimicrobial resistance. Food Res Int. 2024;175:113710. doi: 10.1016/j.foodres.2023.113710 38128981

[98] Zhao K, Liu M, Zhang X, et al. In vitro and in vivo expression of virulence genes in *Trueperella pyogenes* based on a mouse model. Vet Microbiol. 2013;163(3–4):344–350. doi: 10.1016/j.vetmic.2013.01.019 23415031

[99] Risseti RM, Zastempowska E, Twarużek M, et al. Virulence markers associated with *Trueperella pyogenes* infections in livestock and companion animals. Lett Appl Microbiol. 2017;65(2):125–132. doi: 10.1111/lam.12757 28561264

[100] Amos MR, Healey GD, Goldstone RJ, et al. Differential endometrial cell sensitivity to a cholesterol-dependent cytolysin links *Trueperella pyogenes* to uterine disease in cattle. Biol Reprod. 2014 [cited 2014 Mar 13];90(3):54. doi: 10.1095/biolreprod.113.115972 24478394

[101] Jost BH, Lucas EA, Billington SJ, et al. Arcanolysin is a cholesterol-dependent cytolysin of the human pathogen *Arcanobacterium haemolyticum*. BMC Microbiol. 2011 [cited 2011 Oct 26];11(1):239. doi: 10.1186/1471-2180-11-239 22029628 PMC3215231

[102] Liang H, Wang B, Wang J, et al. Pyolysin of *Trueperella pyogenes* induces pyroptosis and IL-1β release in murine macrophages through Potassium/NLRP3/Caspase-1/Gasdermin D pathway. Front Immunol. 2022 [cited 2022 Mar 15];13:832458. doi: 10.3389/fimmu.2022.832458 35371034 PMC8965163

[103] Huang T, Cui K, Song X, et al. MTOR involved in bacterial elimination against *Trueperella pyogenes* infection based on mice model by transcriptome and biochemical analysis. Vet Microbiol. 2019;235:199–208. doi: 10.1016/j.vetmic.2019.06.021 31383303

[104] Fraga AM, Reddy CA. Nutritional requirements of *Corynebacterium pyogenes*. J Clin Microbiol. 1982;16(2):334–340. doi: 10.1128/jcm.162.334-340.1982 6288763 PMC272356

[105] Dong WL, Liu L, Odah KA, et al. Antimicrobial resistance and presence of virulence factor genes in *Trueperella pyogenes* isolated from pig lungs with pneumonia. Trop Anim Health Prod. 2019 Sep;51(7):2099–2103. doi: 10.1007/s11250-019-01916-z 31104226

[106] Ashrafi Tamai I, Mohammadzadeh A, Zahraei Salehi T, et al. Genomic characterisation, detection of genes encoding virulence factors and evaluation of antibiotic resistance of *Trueperella pyogenes* isolated from cattle with clinical metritis. Antonie Van Leeuwenhoek. 2018;111(12):2441–2453. doi: 10.1007/s10482-018-1133-6 30066209

[107] Fujimoto H, Nakamura T, Sato A, et al. Antimicrobial susceptibility of *Truep-erella pyogenes* isolated from cattle and pigs with septicemia in southern Kyushu, Japan. J Vet Med Sci. 2023 Mar 28;85(3):379–382. doi: 10.1292/jvms.22-0460 36775333 PMC10076195

[108] Rzewuska M, Czopowicz M, Gawryś M, et al. Relationships between antimicrobial resistance, distribution of virulence factor genes and the origin of *Trueperella pyogenes* isolated from domestic animals and European bison (Bison bonasus). Microb Pathog. 2016;96:35–41. doi: 10.1016/j.micpath.2016.05.001 27154538

[109] Zastempowska E, Lassa H. Genotypic characterization and evaluation of an antibiotic resistance of *Trueperella pyogenes* (*Arcanobacterium pyogenes*) isolated from milk of dairy cows with clinical mastitis. Vet Microbiol. 2012;161(1–2):153–158. doi: 10.1016/j.vetmic.2012.07.018 22868181

[110] Rzewuska M, Stefańska I, Osińska B, et al. Phenotypic characteristics and virulence genotypes of *Trueperella* (*Arcanobacterium*) *pyogenes* strains isolated from European bison (Bison bonasus). Vet Microbiol. 2012;160(1–2):69–76. doi: 10.1016/j.vetmic.2012.05.004 22658663

[111] Zheng Y, Yu Q, Han L, et al. Molecular characterization of resistance and virulence factors of *Trueperella pyogenes* isolated from clinical bovine mastitis cases in China. Infect Drug Resist. 2024 May 20;17:1979–1986. doi: 10.2147/IDR.S433578 38800580 PMC11122176

[112] Rzewuska M, Kwiecień E, Chrobak-Chmiel D, et al. Pathogenicity and virulence of *Trueperella pyogenes*: a review. Int J Mol Sci. 2019 [cited 2019 Jun 4];20(11):2737. doi: 10.3390/ijms20112737 31167367 PMC6600626

[113] Shan Q, Ma W, Li B, et al. Revealing the mechanism of NLRP3 inflammatory pathway activation through K^+^ efflux induced by PLO via signal point mutations. Int J Mol Sci. 2024 [cited 2024 Jun 18];25(12):6703. doi: 10.3390/ijms25126703 38928408 PMC11203744

[114] Qi M, Liu J, Jiang Q, et al. *Trueperella pyogenes* pyolysin inhibits lipopolysaccharide-induced inflammat-ory response in endometrium stromal cells via autophagy- and ATF6-dependent mechanism. Braz J Microbiol. 2021;52(2):939–952. doi: 10.1007/s42770-021-00422-5 33454924 PMC8105434

[115] Zhang W, Wang H, Wang B, et al. Replacing the 238th aspartic acid with an arginine impaired the oligomerization activity and inflammation-inducing property of pyolysin. Virulence. 2018;9(1):1112–1125. doi: 10.1080/21505594.2018.1491256 30067143 PMC6086297

[116] Rudnick ST, Jost BH, Songer JG, et al. The gene encoding pyolysin, the pore-forming toxin of *Arcanobacterium pyogenes*, resides within a genomic islet flanked by essential genes. FEMS Microbiol Lett. 2003;225(2):241–247. doi: 10.1016/S0378-1097(03)00527-5 12951248

[117] Shan X, Zhao Z, Lai P, et al. RNA nanotherapeutics with fibrosis overexpression and retention for MASH treatment. Nat Commun. 2024 [cited 2024 Aug 27];15(1):7263. doi: 10.1038/s41467-024-51571-8 39191801 PMC11350072

[118] Ross CL, Liang XW, Liu Q, et al. Targeted protein engineering provides insights into binding mechanism and affinities of bacterial collagen adhesins. J Biol Chem. 2012;287(41):34856–34865. doi: 10.1074/jbc.M112.371054 22865854 PMC3464587

[119] Monteiro R, Chafsey I, Caccia N, et al. Specific proteomic identification of collagen-binding proteins in *Escherichia coli* O157: H7: characterisation of OmpA as a potent vaccine antigen. Cells. 2023 [cited 2023 Jun 15];12(12):1634. doi: 10.3390/cells12121634 37371104 PMC10297621

[120] Park SS, Gonzalez-Juarbe N, Martínez E, et al. *Streptococcus pneumoniae* binds to host lactate dehydrogenase via PspA and PspC to enhance virulence. MBio. 2021 [cited 2021 May 4];12(3):e00673–21. doi: 10.1128/mBio.00673-21 33947761 PMC8437407

[121] Ibrahim M, Peter S, Wagener K, et al. Bovine endometrial epithelial cells scale their pro-inflammatory response in vitro to pathogenic *Trueperella pyogenes* Isolated from the bovine uterus in a strain-specific manner. Front Cell Infect Microbiol. 2017 [cited 2017 Jun 21];7:264. doi: 10.3389/fcimb.2017.00264 28680854 PMC5478691

[122] Esmay PA, Billington SJ, Link MA, et al. The *Arcanobacterium pyogenes* collagen-binding protein, CbpA, promotes adhesion to host cells. Infect Immun. 2003;71(8):4368–4374. doi: 10.1128/IAI.71.8.4368-4374.2003 12874314 PMC166022

[123] Pietrocola G, Valtulina V, Rindi S, et al. Functional and structural properties of CbpA, a collagen-binding protein from *Arcanobacterium pyogenes*. Micro-Biol (Read). 2007;153(Pt 10):3380–3389. doi: 10.1099/mic.0.2007/009100-0 17906137

[124] Lőrincz EB, Herczeg M, Houser J, et al. Amphiphilic Sialic acid derivatives as potential dual-specific inhibitors of influenza hemagglutinin and neuraminidase. Int J Mol Sci. 2023 [cited 2023 Dec 8];24(24):17268. doi: 10.3390/ijms242417268 38139095 PMC10743929

[125] Pettigrew MM, Fennie KP, York MP, et al. Variation in the presence of neuraminidase genes among *strept-ococcus pneumoniae* isolates with identical sequence types. Infect Immun. 2006;74(6):3360–3365. doi: 10.1128/IAI.01442-05 16714565 PMC1479257

[126] Soong G, Muir A, Gomez MI, et al. Bacterial neuraminidase facilitates mucosal infection by participating in biofilm production [published correction appears in J Clin Invest. 2006 Oct;116(10): 2828. Kanetko, Yukihiro [corrected to Kaneko, Yukihiro]]. J Clin Invest. 2006;116(8):2297–2305. doi: 10.1172/JCI27920 16862214 PMC1513050

[127] Kim S, Oh DB, Kwon O, et al. Identification and functional characterization of the NanH extracellular sialidase from *Coryne-bacterium diphtheriae*. J Biochem. 2010;147(4):523–533. doi: 10.1093/jb/mvp198 20007980

[128] Shimizu M, Yokoyama T, Sakashita N, et al. Thomsen-friedenreich antigen exposure as a cause of *Streptococcus pyogenes*-associated hemolytic-uremic syndrome. Clin Nephrol. 2012;78(4):328–331. doi: 10.5414/cn107205 22981036

[129] Liu M, Wang B, Liang H, et al. Determination of the expression of three fimbrial subunit proteins in cultured *Trueperella pyogenes*. Acta Vet Scand. 2018 [cited 2018 Sep 12];60(1):53. doi: 10.1186/s13028-018-0407-3 30208923 PMC6134790

[130] Bisinotto RS, Filho JCO, Narbus C, et al. Identification of fimbrial subunits in the genome of *Trueperella pyogenes* and association between serum antibodies against fimbrial proteins and uterine conditions in dairy cows. J Dairy Sci. 2016;99(5):3765–3776. doi: 10.3168/jds.2015-10401 26947291

[131] Abbot EL, Smith WD, Siou GP, et al. Pili mediate specific adhesion of *Streptococcus pyogenes* to human tonsil and skin. Cell Microbiol. 2007;9(7):1822–1833. doi: 10.1111/j.1462-5822.2007.00918.x 17359232

[132] Silva E, Gaivão M, Leitão S, et al. Genomic characterization of *Arcanobacterium pyogenes* isolates recovered from the uterus of dairy cows with normal puerperium or clinical metritis. Vet Microbiol. 2008;132(1–2):111–118. doi: 10.1016/j.vetmic.2008.04.033 18547748

[133] Hobbs M, Dalrymple B, Delaney SF, et al. Transcription of the fimbrial subunit gene and an associated transfer RNA gene of *Pseudomonas aeruginosa*. Gene. 1988;62(2):219–227. doi: 10.1016/0378-1119(88)90560-4 2452767

[134] Doran MH, Baker JL, Dahlberg T, et al. Three structural solutions for bacterial adhesion pilus stability and super-elasticity. Structure. 2023;31(5):529–540.e7. doi: 10.1016/j.str.2023.03.005 37001523 PMC10164138

[135] Jønsson R, Björling A, Midtgaard SR, et al. Aggregative adherence fimbriae form compact structures as seen by SAXS. Sci Rep. 2023 [cited 2023 Oct 2];13(1):16516. doi: 10.1038/s41598-023-42079-0 37783694 PMC10545799

[136] Kwiecień E, Stefańska I, Kizerwetter-Świda M, et al. Genetic diversity and virulence properties of caprine *Trueperella pyogenes* isolates. BMC Vet Res. 2024 [cited 2024 Sep 6];20(1):395. doi: 10.1186/s12917-024-04262-x 39242520 PMC11378509

[137] Moradinezhad M, Abbasi Montazeri E, Hashemi Ashtiani A, et al. Biofilm formation of *Streptococcus mutans*, *Streptococcus sanguinis*, *Staphylococcus epidermidis*, *Staphylococcus aureus*, *Lactobacillus casei*, and *Candida Albicans* on 5 thermoform and 3D printed orthodontic clear aligner and retainer materials at 3 time points: an in vitro study. BMC Oral Health. 2024 [cited 2024 Sep 18];24(1):1107. doi: 10.1186/s12903-024-04893-4 39294648 PMC11412017

[138] Zhao K, Li W, Huang T, et al. Comparative transcriptome analysis of *Trueperella pyogenes* reveals a novel antimicrobial strategy. Arch Microbiol. 2017;199(5):649–655. doi: 10.1007/s00203-017-1338-5 28144921

[139] Alkasir R, Wang J, Gao J, et al. Properties and antimicrobial susceptibility of *Trueperella pyogenes* isolated from bovine mastitis in China. Acta Vet Hung. 2016;64(1):1–12. doi: 10.1556/004.2016.001 26919137

[140] Zhang Z, Liang Y, Yu L, et al. TatD DNases contribute to biofilm formation and virulence in *Trueperella pyogenes*. Front Microbiol. 2021 [cited 2021 Nov 15];12:758465. doi: 10.3389/fmicb.2021.758465 34867886 PMC8634637

[141] Kasimanickam VR, Owen K, Kasimanickam RK. Detection of genes encoding multidrug resistance and biofilm virulence factor in uterine pathogenic bacteria in postpartum dairy cows. Theriogenology. 2016;85(2):173–179. doi: 10.1016/j.theriogenology.2015.10.014 26534827

[142] Mielnichuk N, Joya CM, Monachesi MA, et al. Exopolysaccharide production and preci-pitation method as a tool to study virulence factors. Methods Mol Biol. 2024;2751:71–79. doi: 10.1007/978-1-0716-3617-6_5 38265710

[143] Meneses L, Sillankorva S, Azeredo J. Bacteriophage control of infectious biofilms. Methods Mol Biol. 2024;2734:141–150. doi: 10.1007/978-1-0716-3523-0_9 38066367

[144] Ribeiro MG, Risseti RM, Bolaños CAD, et al. *Trueperella pyogenes* multispecies infections in domestic animals: a retrospective study of 144 cases (2002 to 2012). Vet Q. 2015;35(2):82–87. doi: 10.1080/01652176.2015.1022667 25793626

[145] Zhang D, Zhao J, Wang Q, et al. *Trueperella pyogenes* isolated from dairy cows with endometritis in Inner Mongolia, China: Tetracycline susceptibility and tetracycline-resistance gene distribution. Microb Pathog. 2017;105:51–56. doi: 10.1016/j.micpath.2017.02.010 28188901

[146] Galán-Relaño Á, Gómez-Gascón L, Luque I, et al. Antimicrobial susceptibility and genetic characterization of *Trueperella pyogenes* isolates from pigs reared under intensive and extensive farming practices. Vet Microbiol. 2019;232:89–95. doi: 10.1016/j.vetmic.2019.04.011 31030851

[147] Zhang DX, Tian K, Han LM, et al. Resistance to β-lactam antibiotic may influence *nanH* gene expression in *Trueperella pyogenes* isolated from bovine endometritis. Microb Pathog. 2014;71–72:20–24. doi: 10.1016/j.micpath.2014.04.006 24803199

[148] Yang N, Li H, Yang X, et al. Furazolidone reduces the pathogenesis of *Trueperella pyogenes* and *Pseudomonas aeruginosa* co-infection in a mouse model. Heliyon. 2024 [cited 2024 Oct 19];10(20):e39629. doi: 10.1016/j.heliyon.2024.e39629 39506932 PMC11538771

[149] Lucidi M, Visaggio D, Migliaccio A, et al. Pathogenicity and virulence of *Acinetobacter baumannii*: factors contributing to the fitness in healthcare settings and the infected host. Virulence. 2024;15(1):2289769. doi: 10.1080/21505594.2023.2289769 38054753 PMC10732645

[150] Abdollahi M, Javan AJ, Shokrpoor S, et al. Pyoderma caused by Proteus mirabilis in sheep. Vet Med Sci. 2022;8(6):2562–2567. doi: 10.1002/vms3.926 36049140 PMC9677408

[151] Djebala S, Evrard J, Gregoire F, et al. Antimicrobial susceptibility profile of several bacteria species identified in the peritoneal exudate of cows affected by parietal fibrinous peritonitis after caesarean section. Vet Sci. 2021 [cited 2021 Nov 29];8(12):295. doi: 10.3390/vetsci8120295 34941822 PMC8707031

[152] Kwiecień E, Stefańska I, Chrobak-Chmiel D, et al. *Trueperella pyogenes* isolates from livestock and European Bison (Bison bonasus) as a reservoir of tetracycline resistance determinants. Antibiotics (Basel). 2021 [cited 2021 Apr 3];10(4):380. doi: 10.3390/antibiotics10040380 33916765 PMC8065510

[153] Walker KE, Middleton JR, Gull T, et al. Bacterial culture and susceptibility of samples taken from septic foot lesions of adult beef cattle. J Vet Intern Med. 2023;37(2):757–765. doi: 10.1111/jvim.16645 36772950 PMC10061163

[154] Tamai IA, Mohammadzadeh A, Mahmoodi P, et al. Antimicrobial susceptibility, virulence genes and genomic characterization of *Trueperella pyogenes* isolated from abscesses in dairy cattle. Res Vet Sci. 2023 Jan;154:29–36. doi: 10.1016/j.rvsc.2022.10.018 36434850

[155] Galán-Relaño Á, Gómez-Gascón L, Barrero-Domínguez B, et al. Antimicrobial susceptibility of *Trueperella pyogenes* isolated from food-producing ruminants. Vet Microbiol. 2020;242:108593. doi: 10.1016/j.vetmic.2020.108593 32122597

[156] Yassin Z, Farid A, Ahmadi S, et al. Coronavirus disease 2019 (COVID-19)-associated brain abscesses caused by *Pseudomonas aeruginosa* and *Aspergillus fumigatus*: two case and a review of the literature. J Med Case Rep. 2023 [cited 2023 Dec 4];17(1):520. doi: 10.1186/s13256-023-04206-3 38049820 PMC10694943

[157] Thongrueang N, Liu SS, Hsu HY, et al. An in vitro comparison of antimicrobial efficacy and cytotoxicity between povidone-iodine and chlorhexidine for treating clinical endometritis in dairy cows. PLOS ONE. 2022 [cited 2022 Jul 8];17(7):e0271274. doi: 10.1371/journal.pone.0271274 35802692 PMC9269917

[158] Zhao X, Ding H, Guo A, et al. Zinc(ii)-mediated stereoselective construction of 1,2-cis 2-azido-2-deoxy glycosidic linkage: assembly of *Acinetobacter baumannii* K48 capsular pentasaccharide derivative. Chem Sci. 2024 [cited 2024 Jul 16];15(32):12889–12899. doi: 10.1039/d4sc03449j 39148796 PMC11322977

[159] Wang J, Li W, Li N, et al. Immunization with multiple virulence factors provides maternal and neonatal protection against group B streptococcus serotypes. Vaccines (Basel). 2023 [cited 2023 Sep 5];11(9):1459. doi: 10.3390/vaccines11091459 37766135 PMC10535937

[160] Olaya-Abril A, Gómez-Gascón L, Jiménez-Munguía I, et al. Another turn of the screw in shaving gram-positive bacteria: optimization of proteomics surface protein identification in *Streptococcus pneumoniae*. J Proteomics. 2012;75(12):3733–3746. doi: 10.1016/j.jprot.2012.04.037 22575384

[161] Gómez-Gascón L, Luque I, Tarradas C, et al. Comparative immunosecretome analysis of prevalent *Streptococcus suis* serotypes. Comp Immunol Microbiol Infect Dis. 2018;57:55–61. doi: 10.1016/j.cimid.2018.06.006 30017079

[162] Merrill C, Ensermu DB, Abdi RD, et al. Immunological responses and evaluation of the protection in dairy cows vaccinated with staphylococcal surface proteins. Vet Immunol Immunopathol. 2019;214:109890. doi: 10.1016/j.vetimm.2019.109890 31378218

[163] Żakowska D, Graniak G, Rutyna P, et al. Protective antigen domain 4 of Bacillus anthracis as a candidate for use as vaccine for anthrax. Ann Agric Environ Med. 2019;26(3):392–395. doi: 10.26444/aaem/99669 31559791

[164] Yang L, Liang H, Wang B, et al. Evaluation of the potency of two pyolysin-derived recombinant proteins as vaccine candidates of *Trueperella pyogenes* in a mouse model: pyolysin oligomerization and structural change affect the efficacy of pyolysin-based vaccines. Vaccines (Basel). 2020 [cited 2020 Feb 10];8(1):79. doi: 10.3390/vaccines8010079 32050696 PMC7157609

[165] Lovell R, Foggie A, Pearson JK. Field trials with *Corynebacterium, pyogenes* alum-precipitated toxoid. J Comp Pathol. 1950;60(3):225–229. doi: 10.1016/s0368-1742(50)80021-8 14814228

[166] Machado VS, Bicalho ML, Meira Junior EB, et al. Subcutaneous immunization with inactivated bacterial components and purified protein of *Escherichia coli*, *Fusobacterium necrophorum* and *Trueperella pyogenes* prevents puerperal metritis in Holstein dairy cows. PLOS ONE. 2014 [cited 2014 Mar 17];9(3):e91734. doi: 10.1371/journal.pone.0091734 24638139 PMC3956715

[167] Huang T, Song X, Zhao K, et al. Quorum-sensing molecules N-acyl homoserine lactones inhibit *Trueperella pyogenes* infection in mouse model. Vet Microbiol. 2018;213:89–94. doi: 10.1016/j.vetmic.2017.11.029 29292009

[168] Da Costa RM, Rooke JL, Wells TJ, et al. Type 5 secretion system antigens as vaccines against gram-negative bacterial infections. NPJ Vaccines. 2024 [cited 2024 Sep 1];9(1):159. doi: 10.1038/s41541-024-00953-6 39218947 PMC11366766

[169] Bansal G, Ghanem M, Sears KT, et al. Genetic engineering of *Salmonella spp*. for novel vaccine strategies and therapeutics. EcoSal Plus. [cited 2024 Jul 18];12(1). doi: 10.1128/ecosalplus.esp-0004-2023 PMC11636237 39023252

[170] Jost BH, Songer JG, Billington SJ, et al. An *Arcanobacterium (Actinomyces) pyogenes* mutant deficient in production of the pore-forming cytolysin pyolysin has reduced virulence. Infect Immun. 1999;67(4):1723–1728. doi: 10.1128/IAI.67.4.1723-1728.1999 10085010 PMC96520

[171] Jost BH, Trinh HT, Songer JG, et al. Immunization with genetic toxoids of the *Arcanobacterium pyogenes* cholesterol-dependent cytolysin, pyolysin, protects mice against infection. Infect Immun. 2003;71(5):2966–2969. doi: 10.1128/IAI.71.5.2966-2969.2003 12704180 PMC153263

[172] Pokrajac L, Harris JR, Sarraf N, et al. Oligomerization and hemolytic properties of the C-terminal domain of pyolysin, a cholesterol-dependent cytolysin. Biochem Cell Biol. 2013;91(2):59–66. doi: 10.1139/bcb-2012-0065 23527633

[173] Srivastava IK, Liu MA. Gene vaccines. Ann Intern Med. 2003;138(7):550–559. doi: 10.7326/0003-4819-138-7-200304010-00011 12667025

[174] Galán-Relaño Á, Gómez-Gascón L, Rodríguez-Franco A, et al. Search of potential vaccine candidates against *Trueperella pyogenes* infections through proteomic and bioinformatic analysis. Vaccines (Basel). 2020 [cited 2020 Jun 17];8(2):314. doi: 10.3390/vaccines8020314 32560444 PMC7350218

[175] Ha SJ, Jeon BY, Youn JI, et al. Protective effect of DNA vaccine during chemotherapy on reactivation and reinfection of *Mycobacterium tuberculosis*. Gene Ther. 2005;12(7):634–638. doi: 10.1038/sj.gt.3302465 15690060

[176] Okuda K, Xin KQ, Haruki A, et al. Transplacental genetic immunization after intravenous delivery of plasmid DNA to pregnant mice. J Immunol. 2001;167(9):5478–5484. doi: 10.4049/jimmunol.167.9.5478 11673568

[177] Saha S, Takeshita F, Sasaki S, et al. Multivalent DNA vaccine protects mice against pulmonary infection caused by *Pseudomonas aeruginosa*. Vaccine. 2006;24(37–39):6240–6249. doi: 10.1016/j.vaccine.2006.05.077 16806598

[178] Smith HA, Klinman DM. The regulation of DNA vaccines. Curr Opin Biotechnol. 2001;12(3):299–303. doi: 10.1016/s0958-1669(00)00215-9 11404109

[179] Gurunathan S, Klinman DM, Seder RA. DNA vaccines: immunology, application, and optimization*. Annu Rev Immunol. 2000;18(1):927–974. doi: 10.1146/annurev.immunol.18.1.927 10837079

[180] Huang T, Zhao K, Zhang Z, et al. DNA vaccination based on pyolysin co-immunized with IL-1β enhances host antibacterial immunity against *Trueperella pyogenes* infection. Vaccine. 2016;34(30):3469–3477. doi: 10.1016/j.vaccine.2016.04.025 27091688

[181] Hu Y, Zhang W, Bao J, et al. A chimeric protein composed of the binding domains of *Clostridium perfringens* phospholipase C and *Trueperella pyogenes* pyolysin induces partial immunoprotection in a mouse model. Res Vet Sci. 2016;107:106–115. doi: 10.1016/j.rvsc.2016.04.011 27473983

[182] Cao Y, Bai Y, Li H, et al. Preparation and evaluation of recombinant pyolysin, fimbriae E and HtaA based protein vaccines against *Trueperella pyogenes*. Vet Microbiol. 2023;284:109810. doi: 10.1016/j.vetmic.2023.109810 37307768

[183] Meira EBS Jr, Ellington-Lawrence RD, Silva JCC, et al. Recombinant protein subunit vaccine reduces puerperal metritis incidence and modulates the genital tract microbiome. J Dairy Sci. 2020;103(8):7364–7376. doi: 10.3168/jds.2019-17006 32505392

[184] de Waure C, Gärtner BC, Lopalco PL, et al. Real world evidence for public health decision-making on vaccination policies: perspectives from an expert roundtable. Expert Rev Vaccines. 2024;23(1):27–38. doi: 10.1080/14760584.2023.2290194 38084895

[185] Huang T, Zhao K, Song X, et al. Heterologous prime-boost immunization with DNA Vaccine and modified recombinant proteins enhances immune response against *Trueperella pyogenes* in mice. Vaccines (Basel). 2022 [cited 2022 May 25];10(6):839. doi: 10.3390/vaccines10060839 35746448 PMC9230664

[186] Huang T, Song X, Jing J, et al. Chitosan-dna nanoparticles enhanced the immunogenicity of multivalent DNA vaccination on mice against *Trueperella pyogenes* infection. J Nanobiotechnology. 2018 [cited 2018 Jan 29];16(1):8. doi: 10.1186/s12951-018-0337-2 29378591 PMC5787914

[187] Sircy LM, Ramstead AG, Gibbs LC, et al. Generation of antigen-specific memory CD4 T cells by heterologous immunization enhances the magnitude of the germinal center response upon influenza infection. PLOS Pathog. 2024 [cited 2024 Sep 16];20(9):e1011639. doi: 10.1371/journal.ppat.1011639 39283916 PMC11404825

[188] Zhu J, Paul WE. CD4 T cells: fates, functions, and faults. Blood. 2008;112(5):1557–1569. doi: 10.1182/blood-2008-05-078154 18725574 PMC2518872

[189] Negahdari B, Sarkoohi P, Ghasemi Nezhad F, et al. Design of multi-epitope vaccine candidate based on OmpA, CarO and ZnuD proteins against multi-drug resistant *Acinetobacter baumannii*. Heliyon. 2024 [cited 2024 Jul 16];10(14):e34690. doi: 10.1016/j.heliyon.2024.e34690 39149030 PMC11324976

[190] Waswa EN, Ding SX, Wambua FM, et al. The genus *Actinidia Lindl*. (Actinidiaceae): a comprehensive review on its ethnobotany, phytochemistry, and pharmacological properties. J Ethnopharmacol. 2024;319(Pt 2):117222. doi: 10.1016/j.jep.2023.117222 37793579

[191] Guo Y, Liu Y, Zhang Z, et al. The antibacterial activity and mechanism of action of luteolin against *Trueperella pyogenes*. Infect Drug Resist. 2020 [cited 2020 Jun 10];13:1697–1711. doi: 10.2147/IDR.S253363 32606820 PMC7293968

[192] Zhang L, Cai Y, Li L, et al. Effects of luteolin on biofilm of *Trueperella pyogenes* and its therapeutic effect on rat endometritis. Int J Mol Sci. 2022 [cited 2022 Nov 21];23(22):14451. doi: 10.3390/ijms232214451 36430929 PMC9692790

[193] Guo Y, Huang C, Su H, et al. Luteolin increases susceptibility to macrolides by inhibiting MsrA efflux pump in *Trueperella pyogenes*. Vet Res. 2022 [cited 2022 Jan 10];53(1):3. doi: 10.1186/s13567-021-01021-w 35012652 PMC8744338

[194] Zheng Z, Gao J, Wang C, et al. Genome-wide ide-ntification and expression pattern analysis of the MATE gene family in carmine radish (Raphanus sativus L.). Gene. 2023;887:147734. doi: 10.1016/j.gene.2023.147734 37625557

[195] Zhang D, Gao X, Song X, et al. Luteolin showed a resistance elimination effect on gentamicin by decreasing MATE mRNA expression in silva JCC, Siqueira LC, Rodrigues MX, et al. Intrauterine infusion of a pathogenic bacterial cocktail is associated with the development of clinical metri. Microb Drug Resist. 2019;25(4):619–626. doi: 10.1089/mdr.2018.0097 30431396

[196] Zhang Z, Guo Y, Guo Y, et al. Molecular basis for luteolin as a natural TatD DNase Inhibitor in *Trueperella pyogenes*. Int J Mol Sci. 2022 [cited 2022 Jul 29];23(15):8374. doi: 10.3390/ijms23158374 35955509 PMC9369154

[197] Fu K, Shao L, Mei L, et al. Tanshinone IIA inhibits the lipopolysaccharide- induced epithelial-mesenchymal transition and protects bovine endometrial epithelial cells from pyolysin-induced damage by modulating the nf-κB/Snail2 signaling pathway. Theriogenology. 2021;176:217–224. doi: 10.1016/j.theriogenology.2021.10.001 34628084

[198] Paiano RB, de Sousa RLM, Bonilla J, et al. In vitro effects of cinnamon, oregano, and thyme essential oils against Escherichia coli and *Trueperella pyogenes* isolated from dairy cows with clinical endometritis. Theriogenology. 2023;196:106–111. doi: 10.1016/j.theriogenology.2022.11.010 36413866

[199] Gao F, Fu K, Li H, et al. Chlorogenic acid ameliorates mice clinical endometritis by activating Keap1/Nrf2 and inhibiting NFκB signalling pathway. J Pharm Pharmacol. 2021;73(6):785–795. doi: 10.1093/jpp/rgab020 33734387

[200] Preta G, Lotti V, Cronin JG, et al. Protective role of the dynamin inhibitor dynasore against the cholesterol-dependent cytolysin of *Trueperella pyogenes* europe pmc funders group[j]. 2019. doi: 10.1096/fj.14-265207 PMC4396600 25550455

[201] Barrias ES, Reignault LC, De Souza W, et al. Dynasore, a dynamin inhibitor, inhibits Trypanosoma cruzi entry into peritoneal macrophages. PLOS ONE. 2010 [cited 2010 Jan 20];5(1):e7764. doi: 10.1371/journal.pone.0007764 20098746 PMC2808331

[202] Griffin S, Healey GD, Sheldon IM. Isoprenoids increase bovine endometrial stromal cell tolerance to the cholesterol-dependent cytolysin from *Trueperella pyogenes*. Biol Reprod. 2018;99(4):749–760. doi: 10.1093/biolre/ioy099 29688258 PMC6203874

[203] Huang T, Lv Z, Cui K, et al. Involvement of the E3 ubiquitin ligase Cblb in host defense and evaluation of transcriptome during *Trueperella pyogenes* infection. Microbes Infect. 2023;25(5):105104. doi: 10.1016/j.micinf.2023.105104 36682520

[204] da Silva Duarte V, Dias RS, Kropinski AM, et al. A T4virus prevents biofilm formation by *Trueperella pyogenes*. Vet Microbiol. 2018;218:45–51. doi: 10.1016/j.vetmic.2018.03.025 29685220

[205] Duarte VS, Dias RS, Kropinski AM, et al. Complete genome sequence of vB_ecoM-UFV13, a new bacteriophage able to disrupt *Trueperella pyogenes*. Biofilm Genome Announc. 2016 [cited 2016 Dec 8];4(6):e01292–16. doi: 10.1128/genomeA.01292-16 27932642 PMC5146434

[206] Obisesan AO, Abiodun OO, Ayeni FA. Lactic acid bacteria isolated from women’ breast milk and infants’ faeces have appreciable immunogenic and probiotic potentials against diarrheagenic *E. coli* strains. BMC Microbiol. 2024 [cited 2024 Sep 17];24(1):350. doi: 10.1186/s12866-024-03502-2 39289612 PMC11406810

[207] Liu N, Wu X, Wang M, et al. *Lactobacillus rhamnosus GR-1* ameliorates *Trueperella pyogenes*–induced barrier dysfunction of bovine endometrial epithelial cells. Preprints; 2020. doi: 10.20944/PREPRINTS202010.0185.V1

[208] Ji Y, Song L, Zhou Z, et al. RETRACTED: vB-ApyS-JF1, the first *Trueperella pyogenes* phage, shows potential as an alternative treatment strategy for *Trueperella pyogenes* infections. Front Microbiol. 2023 Dec 01;14:1334746. doi: 10.3389/fmicb.2021.736304 38107865 PMC10722893

[209] Ayadi S, Friedrichs S, Soulès R, et al. 27-Hydroxylation of oncosterone by CYP27A1 switches its activity from pro-tumor to anti-tumor. J Lipid Res. 2023;64(12):100479. doi: 10.1016/j.jlr.2023.100479 37981011 PMC10770617

[210] Ormsby TJR, Owens SE, Horlock AD, et al. Oxysterols protect bovine endometrial cells against pore-forming toxins from pathogenic bacteria. Faseb J. 2021;35(10):e21889. doi: 10.1096/fj.202100036R 34569656 PMC9272411

[211] Civra A, Colzani M, Cagno V, et al. Modulation of cell proteome by 25-hydroxycholesterol and 27-hydroxycholesterol: a link between cholesterol metabolism and antiviral defense. Free Radic Biol Med. 2020;149:30–36. doi: 10.1016/j.freeradbiomed.2019.08.031 31525455 PMC7126780

[212] Hoshina D, Tsujiwaki M, Furuya K, et al. Erythema multiforme associated with *Trueperella pyogenes* bacteremia. J Dermatol. 2017;44(5):e83–e84. doi: 10.1111/1346-8138.13719 27988940

[213] Ormsby TJR, Owens SE, Turner ML, et al. Glucocorticoids increase tissue cell protection against pore-forming toxins from pathogenic bacteria. Commun Biol. 2023 [cited 2023 Feb 17];6(1):186. doi: 10.1038/s42003-023-04568-w 36807406 PMC9938277

[214] Robinson HJ, Phares HF, Graessle OE. Effects of indomethacin on acute, subacute, and latent infections in mice and rats. J Bacteriol. 1968;96(1):6–13. doi: 10.1128/jb.96.1.6-13.1968 5663575 PMC252246

[215] Ashrafi Tamai I, Mohammadzadeh A, Zahraei Salehi T, Mahmoodi P, Pakbin B. Investigation of antimicrobial susceptibility and virulence factor genes in Trueperella pyogenes isolated from clinical mastitis cases of dairy cows. Food Sci Nutr. 2021;9(8):4529–4538. Published 2021 Jun 24. doi: 10.1002/fsn3.2431 34401100 PMC8358342

